# Dynamic functional network reconfiguration underlying the pathophysiology of schizophrenia and autism spectrum disorder

**DOI:** 10.1002/hbm.25205

**Published:** 2020-09-23

**Authors:** Zening Fu, Jing Sui, Jessica A. Turner, Yuhui Du, Michal Assaf, Godfrey D. Pearlson, Vince D. Calhoun

**Affiliations:** ^1^ Tri‐Institutional Center for Translational Research in Neuroimaging and Data Science (TReNDS) Georgia State University, Georgia Institute of Technology, Emory University Atlanta Georgia USA; ^2^ Chinese Academy of Sciences (CAS) Centre for Excellence in Brain Science and Intelligence Technology University of Chinese Academy of Sciences Beijing China; ^3^ Department of Psychology Georgia State University Atlanta Georgia USA; ^4^ School of Computer and Information Technology Shanxi University Taiyuan China; ^5^ Olin Neuropsychiatry Research Center, The Institute of Living Hartford Connecticut USA; ^6^ Department of Psychiatry Yale University School of Medicine New Haven Connecticut USA

**Keywords:** autism spectrum disorder, dynamic functional connectivity, network reconfiguration at different spatial scales, schizophrenia

## Abstract

The dynamics of the human brain span multiple spatial scales, from connectivity associated with a specific region/network to the global organization, each representing different brain mechanisms. Yet brain reconfigurations at different spatial scales are seldom explored and whether they are associated with the neural aspects of brain disorders is far from understood. In this study, we introduced a dynamic measure called step‐wise functional network reconfiguration (sFNR) to characterize how brain configuration rewires at different spatial scales. We applied sFNR to two independent datasets, one includes 160 healthy controls (HCs) and 151 patients with schizophrenia (SZ) and the other one includes 314 HCs and 255 individuals with autism spectrum disorder (ASD). We found that both SZ and ASD have increased whole‐brain sFNR and sFNR between cerebellar and subcortical/sensorimotor domains. At the ICN level, the abnormalities in SZ are mainly located in ICNs within subcortical, sensory, and cerebellar domains, while the abnormalities in ASD are more widespread across domains. Interestingly, the overlap SZ‐ASD abnormality in sFNR between cerebellar and sensorimotor domains was correlated with the reasoning‐problem‐solving performance in SZ (*r* = −.1652, *p* = .0058) as well as the Autism Diagnostic Observation Schedule in ASD (*r* = .1853, *p* = .0077). Our findings suggest that dynamic reconfiguration deficits may represent a key intersecting point for SZ and ASD. The investigation of brain dynamics at different spatial scales can provide comprehensive insights into the functional reconfiguration, which might advance our knowledge of cognitive decline and other pathophysiology in brain disorders.

## Graphical abstract

In this study, we found that both schizophrenia and autism have increased whole‐brain dynamic network reconfiguration and increased dynamic network reconfiguration associated with cerebellar and subcortical/sensorimotor domains. Interestingly, the diseases' overlapping abnormality in dynamic network reconfiguration between cerebellar and sensorimotor domains was correlated with the reasoning‐problem‐solving performance in schizophrenia as well as the Autism Diagnostic Observation Schedule in autism. Our findings suggest that dynamic functional connectivity deficits may represent a key intersecting point for schizophrenia and autism.

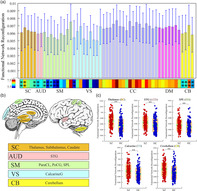

## 1 INTRODUCTION

Evaluating resting‐state brain connectivity from functional magnetic resonance imaging (fMRI) signals has expanded our knowledge of the brain function and neurophysiological mechanisms (Biswal, Zerrin Yetkin, Haughton, & Hyde, 1995; Fornito & Bullmore, 2012; Friston, 2009; Greicius, Krasnow, Reiss, & Menon, 2003). Conventionally, the analysis of resting‐state functional connectivity is based on the assumption that connectivity within a scan session is static, which unfortunately neglects the potential variations of the brain (Hutchison et al., 2013). Research using high‐temporal‐resolution imaging techniques, such as electrophysiological recordings of single cells, local fields, and surface electroencephalograms (EEGs) have observed significant fluctuations of neural activity and connectivity (Lehmann et al., 1998), which indicates that human brain might coordinate different neural populations across multiple spatiotemporal scales to adapt to the internal and external demands (Buzsáki, 2009; Hutchison et al., 2013). Previous studies have reported a wide range of functional connectivity dynamics at different spatial scales, where the spatial scale indicates how the functional organization to be constructed. Those dynamic patterns include temporal variations in local regional homogeneity or specific functional connectivity pairs (Chang & Glover, 2010; Deng, Sun, Cheng, & Tong, 2016), zone of instability of functional networks or domains (Allen et al., 2014; Zalesky, Fornito, Cocchi, Gollo, & Breakspear, 2014), and dynamic graphs and global dynamism of whole‐brain functional connectivity (Fu et al., 2019; Miller et al., 2016; Yu et al., 2015). However, previous work typically used different metrics to investigate the temporal characteristics of different functional organizations. For example, Allen et al. used a metric called zone of instability of functional networks to separate components into groups with more variable functional connectivity (Allen et al., 2014). Global dynamism measures were defined in Miller et al. (2016) for evaluating the dynamic range and fluidity of the whole‐brain. There is a need for a unified measure that can evaluate dynamic patterns of functional brain organizations constructed by different functional connectivity groups. Understanding how functional brain changes at different spatial scales provides more comprehensive information about the macro‐ and micro‐scale spatiotemporal architecture of the brain that is potentially related to human behaviors and brain disorders.

Schizophrenia (SZ) and autism spectrum disorder (ASD) are among many psychiatric and neurological disorders that share overlapping clinical features and similar brain alterations (Konstantareas & Hewitt, 2001). They are both associated with cognitive problems, social withdrawal, and communication impairment, neurodevelopment alterations, and genetic mutations (Association AP, 2013). Early on, they were regarded as the same disorder but in different stages, with ASD manifesting as an earlier phase of SZ (Eisenberg & Kanner, 1956). Individuals with ASD are more likely to have a family history of SZ (Sullivan et al., 2012) and also have increased liability for psychosis (Kincaid, Doris, Shannon, & Mulholland, 2017). Although current diagnostic approaches maintain a nosological distinction between SZ and ASD, their underlying relationship, and neural origins are far from clear. Functional connectivity derived from fMRI is one of the powerful neuroimaging techniques that allow brain configuration abnormalities to be assessed in mental disorders. Studies of brain connectivity have yielded many reliable indicators of brain disorders, with the potential to support early diagnosis and treatment (Arbabshirani, Plis, Sui, & Calhoun, 2017; Greicius, 2008; Koshino et al., 2005; Liu et al., 2008; Whitfield‐Gabrieli et al., 2009). Both SZ and ASD have functional connectivity abnormalities as core disease features reported in previous studies. However, only a few works favor both diseases within a single study, showing most of the common and divergent connectivity alterations in default mode, salience, and motor networks (Chen, Uddin, et al., 2017; Mastrovito, Hanson, & Hanson, 2018; Yoshihara et al., 2020). These studies also have limitations, given that they are based on static connectivity which unfortunately represents a gross oversimplification (Allen et al., 2014). Considering the dynamic nature of the brain, the time‐varying characteristics of functional connectivity are believed to unveil pathophysiology in brain disorders ignored by its static counterpart. Indeed, several existing studies have implied potential similar abnormalities in brain dynamics between SZ and ASD. For example, Damaraju et al. showed that SZ patients have significantly longer dwell times in sparse‐connected states and disrupted thalamocortical connectivity in a state‐specific manner (Damaraju et al., 2014). Similarly, an ASD study found that individuals with ASD have longer dwell time in the weak‐connected states and transient increased thalamic‐sensory connectivity within dynamic states (Fu et al., 2019). The larger variations of dynamic functional connectivity were identified in both SZ (Ma, Calhoun, Phlypo, & Adali, 2014; Yue et al., 2018) and ASD (Chen, Nomi, Uddin, Duan, & Chen, 2017; Harlalka, Bapi, Vinod, & Roy, 2019), showing strong correspondence with the disease‐related hypoconnectivity. The temporal patterns in functional connectivity were suggested to help distinguish SZ from ASD and controls in a recent study (Rabany et al., 2019). The above‐mentioned findings support that studying the dynamic brain reconfiguration for these two disorders, especially from different spatial perspectives is important for clarifying the relationship between SZ and ASD in their brain dynamics, behavioral aspects, and cognitive deficits.

In this study, we first introduce a dynamic measure called step‐wise functional network reconfiguration (sFNR), which is capable of characterizing step‐wise changes in different functional organizations between adjacent time points. The novelty of the current study is twofold. First, our study used the cosine similarity to evaluate the changes in dynamic functional connectivity and then introduced the sFNR based on the step‐wise changes, a unified measure for quantifying the overall temporal characteristics of different functional brain organizations. Second, we applied the sFNR to two diseases' datasets with a large sample size for the exploration of the dynamic abnormalities in different brain organizations that are related to SZ and ASD. We hypothesize that SZ and ASD would have similar but also specific dynamic abnormalities, which are associated with diseases' traits. We adopt a novel analytical framework call Neuromark (Du et al., 2020), combined with sliding‐window correlation and sFNR to two independent datasets, namely the Function Biomedical Informatics Research Network (FBIRN) and the Autism Brain Imaging Data Exchange (ABIDE) to study the abnormal dynamic patterns in SZ and ASD. The relationships between abnormal dynamic reconfiguration and cognitive performance/symptoms are also investigated. The results from the two datasets are then compared to highlight similar and unique changes between SZ and ASD.

## 2 MATERIALS AND METHODS

### 2.1 Datasets

Written informed consent is obtained from all participants of the two independent datasets under protocols approved by the Institutional Review Board. The chracteristics of these two datasets are provided in Table 1. The first dataset is a SZ dataset from the FBIRN. Participants were scanned during the eyes‐closed rest condition. All resting‐state fMRI data were acquired using a standard gradient‐echo echo‐planar imaging (EPI) sequence with TE = 30 ms, TR = 2 s, FA = 77°, slice thickness = 4 mm, slice gap = 1 mm. The duration of each resting‐state scan was 5 min 24 s. Subject inclusion criteria require all participants with head motion <=3° and <=3 mm, and with functional data providing near full brain successful normalization (by comparing the individual mask with the group mask. Details are provided in the supplementary materials). These criteria yield 160 healthy controls (HCs) (average age: 37.04 ± 10.86; range: 19–59 years; 45/115: female/male) and 151 patients with SZ (average age: 38.77 ± 11.63; range: 18–62 years; 36/115: female/male). HCs and SZs are matched by age and gender (age: *p* = .1758; gender: *p* = .3912). HCs do not have any past or current psychiatric illness based on SCID assessment or a first‐degree relative with a diagnosis of an Axis‐I psychotic disorder. SZs are clinically stable at the time of scanning. Most of the participants are measured by a neuropsychological battery that includes six neurocognitive domain tests (speed processing, attention/vigilance, working memory, verbal learning, visual learning, and problem‐solving) called Computerized Multiphasic Interactive Neurocognitive System (CMINDS) (van Erp et al., 2015). CMINDS is a reliable system to evaluate cognitive performance from different angles.

The second dataset is from the release 1.0 of the ABIDE1, a publicly available database sharing resting‐state fMRI data from individuals with ASD and HCs. Before dataset acquisition, consortium members agreed on a “base” phenotypic protocol by identifying overlaps in measures across sites, which include age, sex, IQ, and other diagnostic information. Since in this study, we focus on dynamic variations on functional network connectivity (FNC), we only select subjects with TR = 2 s to avoid inducing confounding effects from different temporal resolutions. The other subject inclusion criteria include criteria for controlling the head motion and normalization quality of data, which are similar to those for the FBIRN dataset. In total 314 HCs (average age: 17.04 ± 7.00; range: 6.47–56.20 years; 65/249: female/male) and 255 individuals with ASD (average age: 17.05 ± 7.74; range: 7.00–55.40 years; 31/224: female/male) from 10 sites are selected in this study. HCs and ASD are matched by age (age: *p* = .9901). Previous literature has demonstrated sexual differentiation for ASD which is far more prevalent in males than in females. There is gender difference (*p* = .0067) on selected HCs and ASD and we thus control for the gender by adding it as a covariate in the statistical analysis.

**TABLE 1:** Demographics FBIRN and ABIDE1 datasets

Characteristics	FBIRN		ABIDE1	
	SZ (*n* = 151)	HCs (*n* = 160)	ASD (*n* = 255)	HCs (*n* = 314)
Age (years)	38.77 ± 11.63	37.04 ± 10.86	17.05 ± 7.74	17.04 ± 7.00
Gender (female/male)	45/115	36/115	31/224	65/249
Problem‐solving	−0.85 ± 1.22	0.04 ± 0.97	NA	NA
ADOS	NA	NA	12.10 ± 3.88 (*n* = 188)	1.25 ± 1.43 (*n* = 28)

Abbreviations: ABIDE1, release 1.0 of the Autism Brain Imaging Data Exchange; ADOS, Autism Diagnostic Observation Schedule; ASD, autism spectrum disorder; FBIRN, Function Biomedical Informatics Research Network; HC, healthy control; SZ, schizophrenia.

### 2.2 Preprocessing

The fMRI data were preprocessed using the statistical parametric mapping (SPM12, http://www.fil.ion.ucl.ac.uk/spm/) package in MATLAB 2016 environment. The first five scans were removed for the signal equilibrium and participants' adaptation to the scanner's noise. We performed rigid body motion correction using the toolbox in SPM to correct subject head motion, followed by the slice‐timing correction to account for timing difference in slice acquisition. The fMRI data were subsequently warped into the standard Montreal Neurological Institute space using an EPI template and were slightly resampled to 3 × 3 × 3 mm^3^ isotropic voxels. The resampled fMRI data were further smoothed using a Gaussian kernel with a full width at half maximum = 6 mm. After the preprocessing, the FBIRN subjects have 157 scans and the ABIDE1 subjects have 145 scans used in the following analysis.

### 2.3 Functional network extraction

To obtain the same functional network structure for both datasets, a set of network priors were first extracted by the Neuromark framework (Du et al., 2020). In this framework, spatial group ICA was performed on two independent datasets with a large sample of HCs (Human Connectome Project [HCP, 823 subjects after the subject selection] and Genomics Superstruct Project [GSP, 1005 subjects after the subject selection]). We choose these two datasets with different temporal resolutions and preprocessed by different pipelines because we would like to identify network priors that are consistent and reproducible across various conditions.

For each dataset, principal component analysis was performed to reduce the subject‐specific data into 120 principal components (PCs) which preserve more than 95% variance of the original data. The 120 PCs of each subject were concatenated across subjects and then reduced into 100 PCs at the group level. The infomax ICA algorithm was conducted to decompose the 100 PCs into 100 independent components (ICs) and such procedure was repeated 10 times in ICASSO, in which the best run was selected to ensure the estimation stability. The estimated ICs from the two datasets were then matched by comparing their corresponding group‐level spatial maps. A spatial similarity matrix S (size: 100 × 100) was obtained by computing the absolute value of Pearson correlation coefficients between spatial maps of ICs from GSP and that from HCP. Based on the matrix S, the pair of ICs with the maximum correlation value was selected and considered as the first‐matched components pair. If their original correlation value was negative, one of the ICs was sign‐flipped. After identifying a matched ICs pair, the correlation values related to them in matrix S were set to zero, resulting in a new similarity matrix S_new_. As such, the matching procedure was repeated continually on the updated correlation matrices, until the final matched IC pair was found. IC pairs were considered to be reproducible if they show a higher spatial correlation than a given threshold. Previous studies have shown that a correlation threshold = 0.25 indicates a significant correspondence (*p* < .005, corrected) between components (Beckmann, DeLuca, Devlin, & Smith, 2005; Beckmann, Mackay, Filippini, & Smith, 2009). In our study, we used a larger threshold (=0.4) which promoted higher correspondence between the matched ICs from HCP and GSP. We characterized a subset of these matched ICs as ICNs, as opposed to physiological, movement‐related, or imaging artifacts. Components were evaluated by three experts' votes, which are based on the expectations that ICNs should have their activation peaks fell on gray matter and low spatial over‐lap with known vascular, ventricular, motion and some other artifacts, and should have dominant low‐frequency fluctuations on their corresponding time‐courses (TCs).

We used the less noisy ICNs captured from the GSP dataset (note that there are 53 ICNs from HCP which have similar spatial patterns) as the spatial network priors applied group‐information‐guide ICA (gig‐ICA) to back‐reconstructed subject‐specific spatial maps and TCs for the FBIRN and ABIDE1 datasets. Via using the Neuromark framework, the identified ICNs from FBIRN and ABIDE1 are corresponding, leveraging the feasibility of obtaining the same network structure across datasets. After extracting ICNs and their corresponding TCs for each dataset, ICN TCs underwent additional postprocessing to remove the remaining noise. These procedures included (a) detrending linear, quadratic, and cubic trends; (b) multiple regression of the six realignment parameters and their derivatives, (c) removal of detected outliers, and (d) low‐pass filtering with a cutoff frequency of 0.15 Hz.

### 2.4 Dynamic functional network connectivity

For each subject, dynamic FNC (dFNC) was estimated by a sliding window approach. A tapered window, created by convolving a rectangle (width = 20 TRs = 40 s, TR = 2 s) with a Gaussian (*σ* = 3 TRs), was used to segment the TCs. We slid the window in steps of 1 TR, resulting in total *W* = 137 windows for the FBIRN subjects and *W* = 125 for the ABIDE subjects. It is suggested that window size between 30 s and 1 min produces reliable dynamic functional connectivity estimates (Hutchison et al., 2013). Robust dynamic functional connectivity patterns have been well identified using the sliding window approach with a similar window (Allen, Damaraju, Eichele, Wu, & Calhoun, 2018; Fiorenzato et al., 2019; Fu et al., 2019; Hutchison et al., 2013; Tu et al., 2019; Zalesky et al., 2014). To further show the reliability of our results, we also calculated the dFNC using a wide range of window sizes (16–24 TRs) and provided the results in the supplementary materials. Considering that the short time segment may not have sufficient information to characterize the full covariance matrix, we estimate covariance from the regularized precision matrix, which is calculated via the graphical LASSO method (Friedman, Hastie, & Tibshirani, 2008) on the windowed data. The regularization parameter was optimized for each subject by using a cross‐validation framework.

### 2.5 Calculation of sFNR

Consider two matrices A and B with dimension N × M, where each entry represents the link between nodes. The distance between these two matrices can be defined as:

(1) $$\delta \left(A,B\right)=1-\vartheta \left(A,B\right).$$

where *ϑ* is the cosine similarity function calculating as (Han & Goetz, 2019):

$$\vartheta \left(A,B\right)=\frac{J\left(A\;\circ \;B\right)J^{T}}{{\left\|A\right\|}_{F}{\left\|B\right\|}_{F}},\text{if}\;N=M;$$

(2) $$\vartheta \left(A,B\right)=\frac{A\left(:\right)\;\circ \;B\left(:\right)}{{\left\|A\right\|}_{E}{\left\|B\right\|}_{E}},\text{if}\;N\neq M.$$

here *J* is a 1 × N vector with 1 for each element and ∘ is the Hadamard product. ‖‖_*F*_ is the Frobenius norm and ‖‖_*E*_ is the Euclidean norm. Therefore, if A presents a network of previous time point (*t*‐1) and *B* represents a network of current time point (*t*), the sFNR of this time point (*t*) can be measured by the distance between them. The sFNR *δ* is calculated for each time point to evaluate how the functional network reconfigures time by time (so called step‐wise). In addition, sFNR is within the scope 0–1, allowing us to compare it across different networks and across groups, where a larger value of *δ* indicates the greater network reconfiguration.

The dFNC of each window were concatenated to form a C × C × W array (C: number of ICNs; W: number of windows), representing the changes in brain connectivity as a function of time. We calculated sFNR of brain for: (a) whole‐brain, based on C × C × W dFNC array; (b) domain, based on C_i_ × C_j_ × W dFNC array, where C_i_ × C_j_ of each time window presents FNC between domains i and j or within domain i if i = j; and (c) ICN, based on 1 × (C‐1) × W dFNC array, where 1 × (C‐1) represents FNC between a given ICN and the other ICNs. For each subject, the outputs of this procedure are a (W‐1) × 1 sFNR vector for the whole‐brain, a L × (W‐1) sFNR array for the domain level where L = (D + 1) × D ÷ 2 (D denotes the number of domain), and a C × (W‐1) sFNR array for the ICN level. The output sFNR is then averaged across windows (W‐1) to obtain the overall FNR during the resting‐state. sFNR is then averaged across windows for further analysis. A general linear model (GLM) was applied to examine whether the average sFNR shows differences between SZ and HC, controlling for age and gender. The GLM was also applied to examine the group difference between ASD and HC on the average sFNR, controlling for age, gender, and site. Different from the FBIRN data that were scanned using the same structural and functional scan parameters (e.g., data length, TR/TE, and slice order), the data from ABIDE are scanned using different protocols which could result in more systematic differences between subjects from different sites. Therefore, for controlling for the multisites effect, we generated a Subject × (*N*‐1) array as the input covariate in the GLM analysis, where the N is supposed to be the total number of sites in this data. For each column i = 1, 2, …, *N*‐1, if a subject is from site i, the entry (sub, *i*) of this array is set as 1, otherwise, the entry would be set as 0. To further show that the site information in the FBIRN data would not influence our findings, we also controlled for the site effect in the statistical analysis between SZ and HCs and provided the results in the supplementary materials. To further investigate the associations between the atypical sFNR and cognitive performance and symptoms, the GLM was used to examine the association between abnormal sFNR and CMIND scores for the FBIRN dataset and the association between abnormal sFNR and the Autism Diagnostic Observation Schedule (ADOS) for the ABIDE1 dataset, controlling for additional diagnosis effect. Statistical results were corrected using Bonferroni correction (Dunnett, 1955) or false discovery rate (FDR) correction (Benjamini & Hochberg, 1995).

## 3 RESULTS

### 3.1 Neuromark ICNs

Then, 53 ICNs were identified by the Neuromark pipeline, with the activation peaks fell on the majority of subcortical and cortical gray matter. According to prior functional and anatomical information, ICNs are arranged into seven functional domains: subcortical (SC), auditory (AUD), visual (VS), sensorimotor (SM), cognitive‐control (CC), default‐mode (DM), and cerebellar (CB) domains. The spatial maps, component labels, and peak coordinates of ICNs are provided in the supplementary material.

### 3.2 Increased sFNR in SZ

**FIGURE 1:**
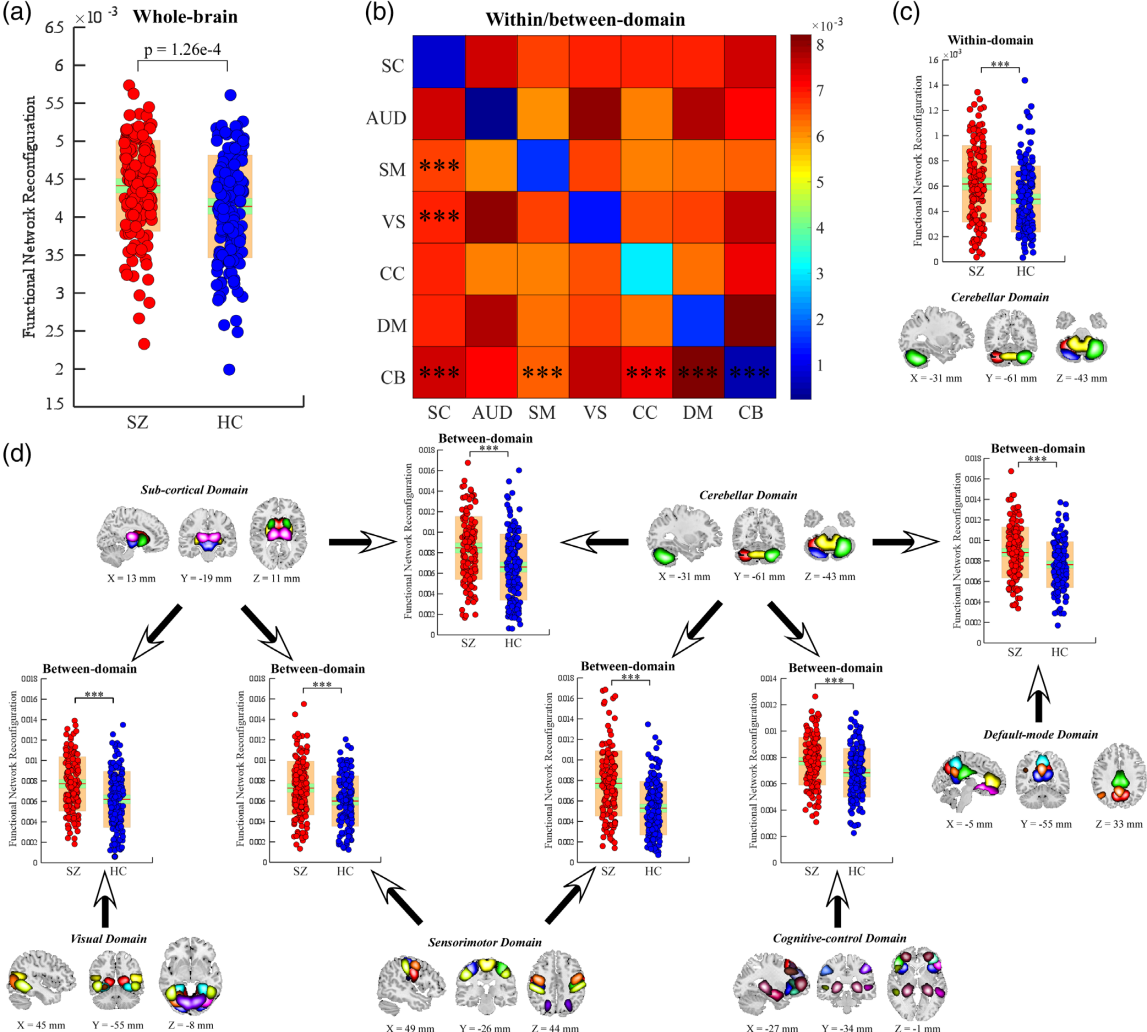
sFNR abnormalities in SZ. (a) Difference in the whole‐brain sFNR between HCs and SZ. (b) Average within/between‐domain sFNR across all subjects. (c) Difference in within‐domain sFNR between HC and SZ. (d) Difference in between‐domain sFNR between HC and SZ. Boxplots display the mean sFNR across subjects (red line), the 95% confidence interval for the mean (green area), and the *SD* (orange area). ***Significance *p* < .01, Bonferroni corrected. HC, healthy control; sFNR, step‐wise functional network reconfiguration; SZ, schizophrenia

**FIGURE 2:**
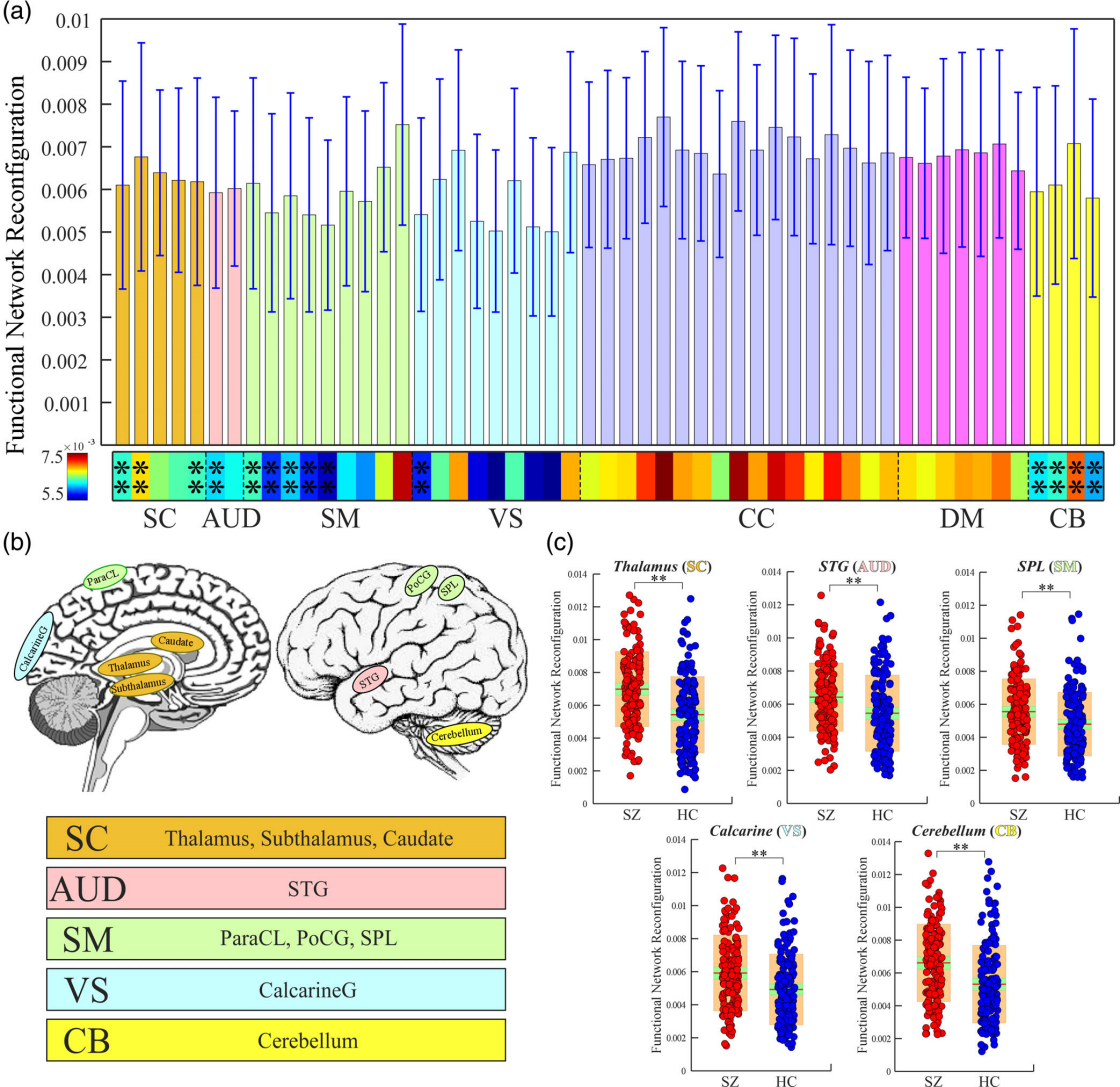
sFNR abnormalities for ICNs in SZ patients. (a) sFNR across ICNs. (b) Highlighted ICNs with sFNR difference between HCs and SZ patients. (c) Exemplar ICNs with sFNR difference from five different functional domains. Boxplots display the mean sFNR across subjects (red line), the 95% confidence interval for the mean (green area), and the *SD* (orange area). **Significance *p* < .05, Bonferroni corrected. HC, healthy control; ICN, intrinsic connectivity network; sFNR, step‐wise functional network reconfiguration; SZ, schizophrenia

Compared with HC, SZ has increased sFNR of the brain at different scales. Specifically, sFNR of the whole‐brain significantly increases in SZ (*p* = 1.26 e‐4; Figure 1). Within‐domain sFNR is smaller than between‐domain sFNR, suggesting more nonstationary properties in the FNC between different functional modules. Greater sFNR are identified between SC and SM/VS/CB domains, between CB and SM/CC/DM domains and within CB domains (***significance *p* < .01, Bonferroni corrected; Figure 1) in SZ. The SC, SM, and CB domains are the dominant functional modules that are associated with increased sFNR in SZ. For the sFNR at the ICN level, ICNs within CC domain are less stable while the ICNs within sensory domains (AUD, SM, and VS) are more stationary. However, it could be also observed increased sFNR in several sensory ICNs, indicating more nonstationary relationships between them and the other ICNs. For example, the postcentral gyrus (PoCG) and the middle temporal gyrus (MTG) have a relatively higher degree of sFNR (Figure 2). ICNs within SC domain (including caudate, subthalamus, thalamus), AUD domain (including superior temporal gyrus [STG]), SM domain (including PoCG, paracentral lobule [ParaCL], superior parietal lobule [SPL]), VS domain (calcarine gyrus), and CB domain show greater sFNR in SZ (**significance *p* < .05, Bonferroni corrected; Figure 2). Most abnormalities are concentrated in SC, CB, and sensory domains.

### 3.3 Increased sFNR in ASD

**FIGURE 3:**
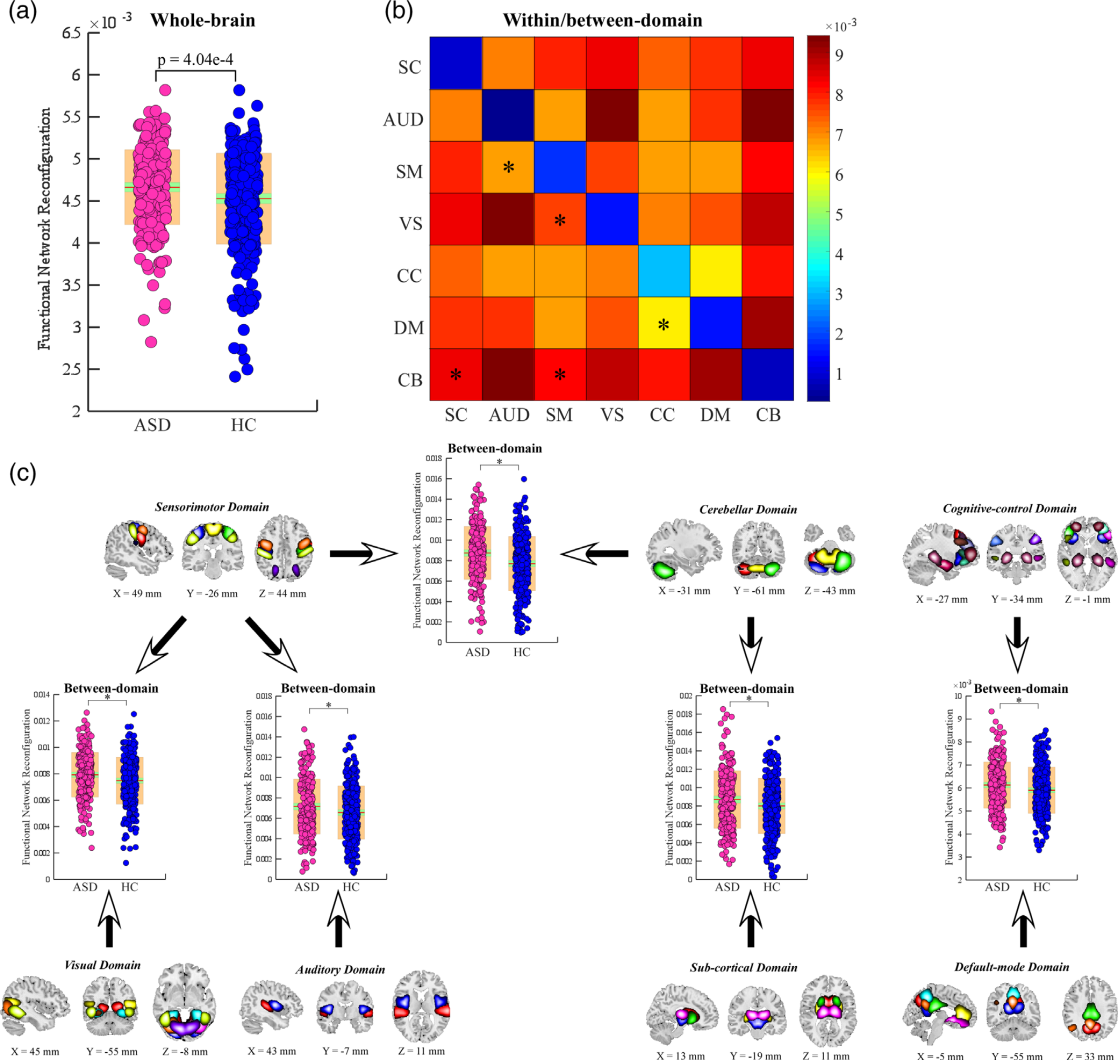
sFNR abnormalities in ASD. (a) Difference in the whole‐brain sFNR between HCs and individuals with ASD. (b) Average within/between‐domain sFNR across all subjects. (c) Difference in between‐domain sFNR between HC and ASD. Boxplots display the mean sFNR across subjects (red line), the 95% confidence interval for the mean (green area), and the *SD* (orange area). *Significance *p* < .05, FDR corrected. ASD, autism spectrum disorder; FDR, false discovery rate; HC, healthy control; sFNR, step‐wise functional network reconfiguration

**FIGURE 4:**
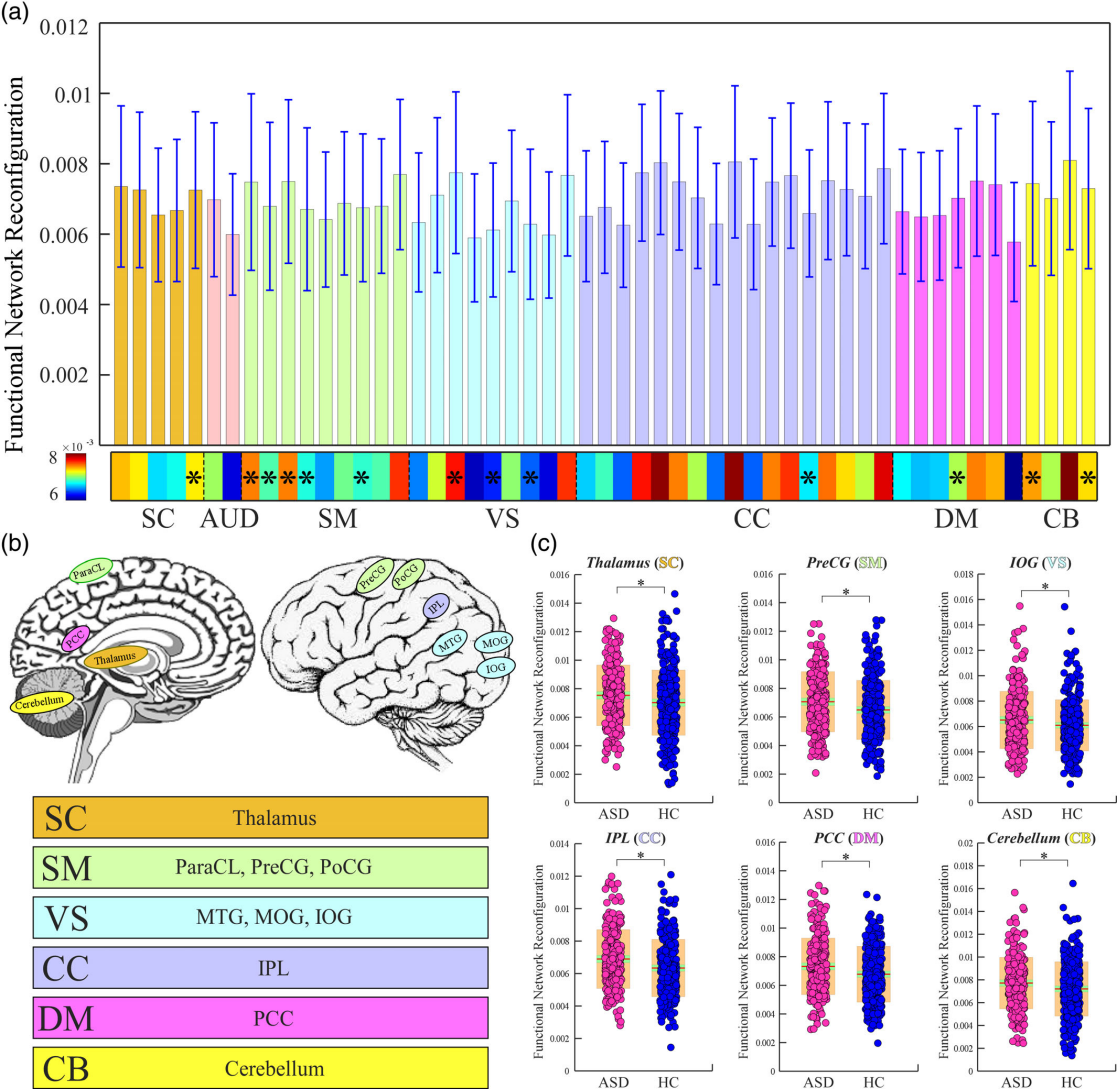
sFNR abnormalities for ICNs in ASD patients. (a) sFNR across ICNs. (b) Highlighted ICNs with sFNR difference between HCs and individuals with ASD. (c) Exemplar ICNs with sFNR difference from six different functional domains. Boxplots display the mean sFNR across subjects (red line), the 95% confidence interval for the mean (green area), and the *SD* (orange area). *Significance *p* < .05, FDR corrected. ASD, autism spectrum disorder; FDR, false discovery rate; HC, healthy control; ICN, intrinsic connectivity network; sFNR, step‐wise functional network reconfiguration

Similar sFNR changes are identified in ASD. Specifically, individuals with ASD show a higher degree of whole‐brain sFNR (*p* = 4.04 e‐4; Figure 3). The whole‐brain sFNR is associated with ADOS (*p* = .0136). At the domain level, within‐domain sFNR is relatively smaller than between‐domain sFNR, which is consistent with the findings from FBIRN. Individuals with ASD show similar alterations in sFNR between CB and SC/SM domains, but the significance level is weaker (*significance *p* < .05, FDR corrected; Figure 3). ASD also has specific sFNR changes, mainly between SM and AUD/VS and between CC and DM (*significance *p* < .05, FDR corrected; Figure 3). Similar to SZ, the SM and CB are the most affected functional domains. However, the SC domain is less affected in ASD. The sFNR of ICNs shows similar distribution patterns in both FBIRN and ABIDE1 datasets. For instance, ICNs within CC have relatively higher sFNR. PoCG and MTG have higher sFNR compared with the other ICNs within the sensory domains (Figure 4). Different from the SZ, individuals with ASD have altered sFNR widespread across domains. ICNs within SC domain (thalamus), SM domain (precentral gyrus [PreCG], PoCG, and ParaCL), VS domain (MTG, middle occipital gyrus [MOG], and inferior occipital gyrus [IOG]), CC domain (inferior parietal lobule [IPL]), DM domain (posterior cingulate cortex) and CB domain show greater sFNR in ASD (*significance *p* < .05, FDR corrected; Figure 4).

### 3.4 Associations between sFNR and cognitive deficits and autistic traits

**FIGURE 5:**
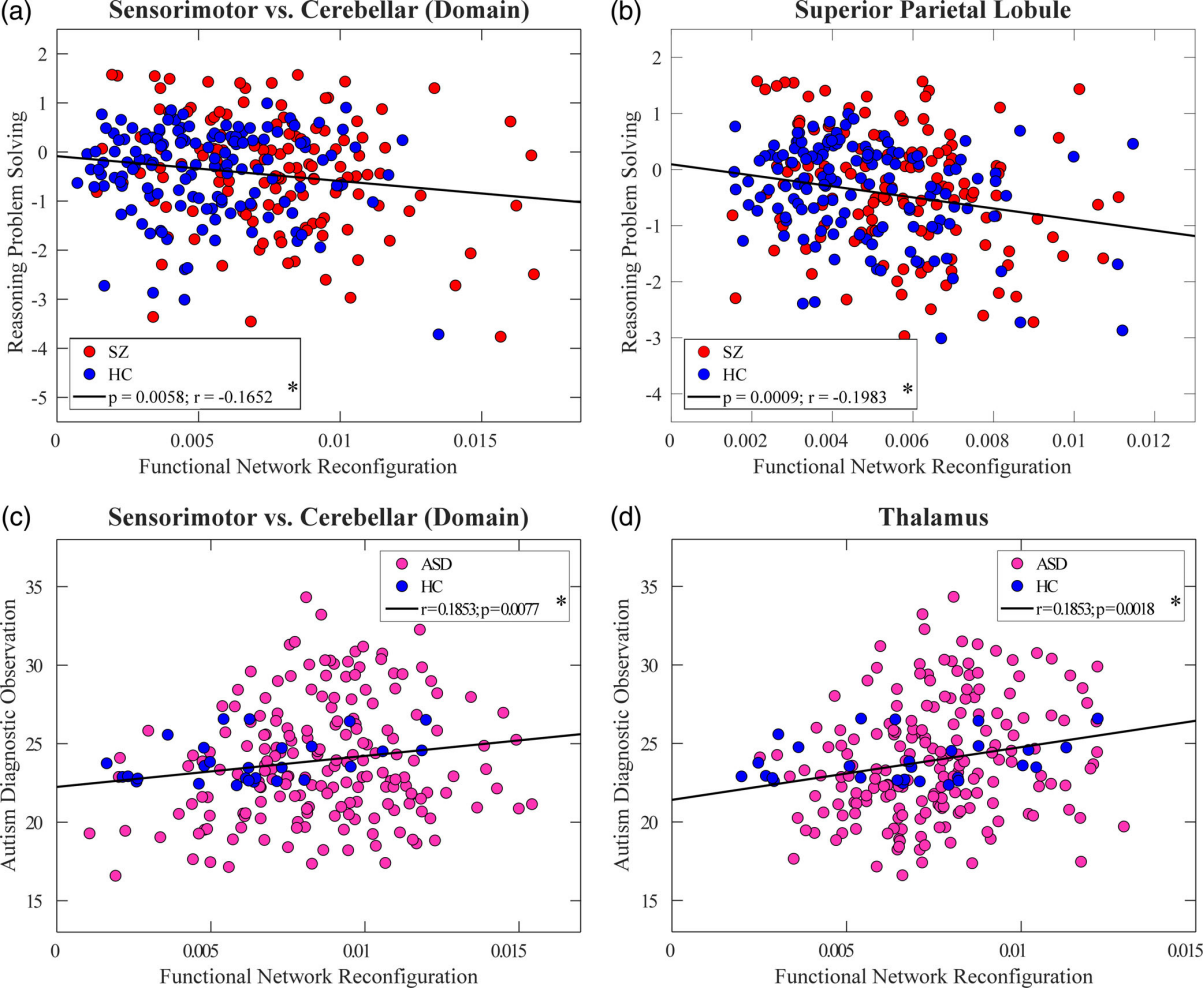
Correlations between sFNR and reasoning‐problem‐solving score/ADOS. Two sFNR calculations show negative correlations with reasoning‐problem‐solving and two sFNR calculations show positive correlations with ADOS: (a) sFNR between SM and CB domains in SZ; (b) sFNR of SPL in SZ; (c) sFNR between SM and CB domains in ASD; and (d) sFNR of thalamus in ASD. Each dot represents the value of each subject and the black line represents the relationship between sFNR and score. *Significance *p* < .05, FDR corrected. ADOS, the Autism Diagnostic Observation Schedule; ASD, autism spectrum disorder; CB, cerebellar domain; sFNR, step‐wise functional network reconfiguration; SM, sensorimotor domain; SPL, superior parietal lobule; SZ, schizophrenia

We further found significant associations between atypical sFNR and cognitive performance in the FBIRN dataset. The score of reasoning‐problem‐solving is negatively correlated with the sFNR between SM and CB domains and the sFNR of SPL (*significance *p* < .05, FDR corrected; Figure 5), indicating that the poorer cognitive performance in SZ is accompanied with the larger sFNR. An interesting patterns between SZ and ASD is that the sFNR between SM and CB domains is also positively correlated with ADOS in ASD (*significance *p* < .05, FDR corrected; Figure 5). We also found a significant association at the ICN level (ADOS is associated with the sFNR of thalamus, *significance *p* < .05, FDR corrected), as compared with the findings in SZ (cognitive performance is associated with the sFNR of SPL). We also correlated the abnormal sFNC measures with the head motion parameter as measured by the mean framewise displacement and there is no significant correlation between them (*p* > .05), indicating that such abnormalities are not due to the potential confounding effects from head motion.

### 3.5 Overlapping and unique abnormalities in SZ and ASD

At the whole‐brain level, both SZ and ASD show increased functional reconfiguration compared with HCs. At the within/between‐domain level, SZ and ASD have overlapping abnormalities as increased functional reconfiguration between CB and SC domains, and between CB and SM domains. SZ has unique abnormal sFNR between SC and SM/VS domains, between CB and CC/DM domains, and within CB domain. ASD has unique abnormalities in sFNR between SM and AUD/VS domains and between CC and DM domains. At the ICN level, although the abnormalities in ASD are more wide‐spread, SZ and ASD share overlapping sFNR abnormalities related to the ICNs mainly located in the SC, SM, and CB domain, consistent with the results at the domain level. Increased sFNR identified in both diseases is involved in thalamus, left and right PoCG, ParaCL, and cerebellum. SZ shows a unique increase in sFNR related to caudate, subthalamus, STG, SPL, and calcarine gyrus while ASD shows a unique increase in sFNR related to PreCG, MTG, MOG, IOG, and left IPL.

## 4 DISCUSSION

In this study, we found that the functional brain reconfiguration significantly differs between groups, being higher in ASD and SZ patients. Functional domains with a higher degree of reconfiguration are mainly associated with sensorimotor and cerebellar systems. Interestingly, the increased reconfiguration in both diseases is associated with more sever symptom traits as well as poorer cognitive performance. Collectively, our findings suggest that characterizing the brain reconfiguration at different spatial scales might be a crucial piece in providing a comprehensive picture of brain dynamics.

### 4.1 Abnormal whole‐brain network reconfiguration

Network rewiring measures have been successfully employed to multiple networks and detect reliable network reconfigurations (Han & Goetz, 2019). Existing approaches, like Dynamic Graphical Model (DGM) (Schwab et al., 2018) and Group Iterative Multiple Model Estimation (GIMME) (Gates & Molenaar, 2012), have been widely used in fMRI studies to capture the connectivity structure during the resting‐state. However, there are two major differences between our method and these approaches. First, DGM and GIMME are used to describe instantaneous directed relationships between brain regions. They are more focusing on effective connectivity, which indicates the causal relationships between brain regions with systematic lags. Instead, our method is focusing on the undirected functional connectivity, which is represented by the statistical dependence between brain regions without any lags. Second, DGM and GIMME do not consider any time‐varying experimental conditions or designed input and favor only constant or consistent relationships between brain regions during the whole scan. In contrast, our method evaluates how a group of functional connectivity varies time by time and is capable of quantifying the temporal characteristics of different functional brain organizations. Multiple lines of evidence suggest the existence of covarying patterns in functional connectivity (Allen et al., 2014; Liu & Duyn, 2013; Liu, Zhang, Chang, & Duyn, 2018), which indicates that measuring the dynamics in each functional connectivity independently and simply averaging them across functional connectivity might not enough to capture the real dynamic fluctuations of a functional organization constructed by a group of functional connectivity. Compared with the metrics like the *SD* of sliding‐window correlation and nonstationarity test statistic in (Zalesky et al., 2014), the sFNR evaluates the changes of a group of functional connectivity simultaneously and is capable of capturing the global dynamism of functional organization directly. The sFNR also shows advantages to the clustering‐based approaches, which assume that certain connectivity patterns may reoccur over time and are present in numerous subjects (Allen et al., 2014). The difference between the number of clusters and even between the brain state patterns due to the use of the clustering‐based approaches makes it difficult to compare findings across studies or diseases. In contrast, by combining with the Neuromark framework, the sFNR can be easily applied to different data with the feasibility of comparing findings from different datasets without the need for matching the dynamic brain states. In addition, the sFNR can evaluate the dynamic properties at different spatial scales, while the clustering‐based approaches are typically used to capturing the dynamic characteristics of the whole‐brain.

Using the average sFNR, we found that SZ has larger whole‐brain reconfiguration. Functional dysconnectivity has been widely reported in SZ but the root cause is still unclear. The finding of larger functional network variability indicates temporal disorganization of sequentially expressed dynamic connectivity, which might be a potential cause of the formulations of dysconnectivity (Breakspear & Stam, 2005). Our observed increased relatively high‐frequency (step‐wise) network reconfiguration might be supported by a previous study from a high‐temporal resolution technique magnetoencephalography (Siebenhühner, Weiss, Coppola, Weinberger, & Bassett, 2013). They showed that during a two‐back working memory task, SZ patients exhibit more network topology variability across trials. Resting‐state is a unconstrained condition which might contain different mental processes associating with different connectivity patterns (Allen et al., 2014; Marusak et al., 2017). Each mental process can be regarded as a single activity trail and SZ patients might also have increased network reconfiguration/variability in the resting‐state as they did in the memory task. Similar dynamic patterns are observed in an EEG study, in which an increased entropy of high‐frequency connectivity are identified in SZ (Schoen, Chang, Lee, Bob, & Mashour, 2011). Larger whole‐brain reconfiguration in ASD is observed in our study as well, indicating overlapping dynamic abnormalities between ASD and SZ. Higher temporal variability of pair‐wise functional connectivity has been previously identified in ASD (Chen, Nomi, et al., 2017; Falahpour et al., 2016). Our results are consistent with these findings and further indicate an increased global reconfiguration of functional brain. The greater network reconfiguration might provide evidence that the overall brain network is not actually “disrupted” in both diseases but exhibit more unstable patterns over time.

The increased whole‐brain reconfiguration in SZ and ASD can also be explained by the excessive neural variability in brain disorders (Markram & Markram, 2010; Simmons et al., 2009). Excessive trial‐to‐trial variability of brain responses is widely observed in both diseases, which are suggested to be strong evidence of excessive neural variability in brain disorders (Dinstein et al., 2010; Dinstein et al., 2012; Siebenhühner et al., 2013). We argue that the larger brain network reconfiguration might be an alternative representation of excessive neural variability. The highly fluctuated brain organization would result in unstable communications between brain areas, so that influences the perception of the environment (Dinstein, Heeger, & Behrmann, 2015). As such, patients may have difficulties to interact with external stimuli and therefore exhibit specific behavioral symptoms and cognitive decline.

### 4.2 Abnormal network reconfiguration in functional domains and ICNs

Abnormal functional reconfiguration was identified in functional domains and ICNs in both disorders. Specifically, SZ shows increased reconfiguration within and between subcortical, sensory and cerebellar domains. The more nonstationary patterns in the functional connectivity between subcortical and cortical regions in SZ might indicate the inability of the brain to sustain the connections between subcortical‐sensory‐cerebellar domains necessary for healthy cognitive function, which might empirically result in the fragmentation of cortical and subcortical networks seen in patients (van den Berg, Gong, Breakspear, & van Leeuwen, 2012).

Abnormal brain connectivity is one of the major pathophysiological mechanisms of SZ (Bullmore, Frangou, & Murray, 1997). Numerous brain regions involved in temporal, parietal, subcortical, and cerebellar networks and the associated functional connectivity have been linked to SZ and their alterations are suggested to play vital roles in the pathophysiology of this disease (Jafri, Pearlson, Stevens, & Calhoun, 2008; Jones et al., 2012; Ma et al., 2014). Our results provide supports for these findings and further suggest the importance of evaluating the temporal changes in these brain networks and their connectivity. Increased spatial diversity and complexity of the functional organization are also key abnormities in SZ, being highly sensitive to the disease state (Bassett, Nelson, Mueller, Camchong, & Lim, 2012; Lynall et al., 2010). The higher network reconfiguration observed in our study might be complementary results to these previous findings by revealing the increased temporal diversity of functional organization in SZ. Besides, the observed greater network reconfiguration between a single ICN and the rest of the brain seems compatible with the reduced homogeneity of regional activity found in SZ (Liu et al., 2006), given that the regions with dissimilar activities might have weaker and more nonstationary connectivity, resulting in higher network variability.

Similar but weaker brain alterations were identified in ASD in our study. Our results are in line with the observations that both SZ and ASD displayed atypical temporal patterns, but the SZ showed more pervasive abnormalities (Rabany et al., 2019). The domain level sFNR abnormalities in ASD are mainly associated with sensorimotor and cerebellar domains. The increased cerebral activity but decreased cerebellar activity in ASD has been documented in literature (Mostofsky et al., 2009; Müller, Kleinhans, Kemmotsu, Pierce, & Courchesne, 2003). One possible explanation of such cerebral‐cerebellar dissociation can be the underconnectivity between those brain regions (Mostofsky et al., 2009). Our findings suggest that the more variable connectivity between these functional domains might be another potential cause of such cerebral‐cerebellar dissociation. That is, given the more unstable communication between the cerebellum and sensorimotor areas, their information exchange will be less reliable. Therefore, it may be more efficient for ASD to utilize these regions as independent processors, rather than having them working together (Mostofsky et al., 2009). The results of ICN sFNR indicate different patterns of abnormalities between SZ and ASD. Compared with SZ, ASD has atypical reconfiguration on ICNs widespread domains. We speculated that is because ASD influences more variety of brain systems, which might be related to the more diverse social skill, attention switching and communication problem in this disease (Spek & Wouters, 2010).

### 4.3 Correlation between network reconfiguration and cognition/symptoms

In this study, we characterized convergent and divergent brain abnormalities in SZ and ASD which are associated with the cognitive decline and autistic symptoms. On the one hand, higher network reconfiguration between sensorimotor and cerebellar domains in both diseases is correlated with reasoning problem‐solving from the SZ dataset and ADOS from the ASD dataset. Although the sensory and cerebellar domains were previously regarded to be involved in sensory and motor functions, there is a wide recognition that they are also associated with cognitive processing and behaviors (Cerliani et al., 2015; Schmahmann & Caplan, 2006). Cerebellum, which was considered as a coordinator of motor function for many years, has been shown to also play an important role in cognitive functions and emotion processing based on the evidence from anatomical, clinical, and neuroimaging data (Schmahmann & Caplan, 2006; Stoodley, 2012). The functional connectivity abnormalities in sensory networks are also suggested to be linked to the difficulties in inhibiting repetitive behaviors in ASD (Cerliani et al., 2015). Anatomical and physiological studies show that the cerebellum receives information from the sensory systems, indicating a strong interaction between the cerebellar and sensorimotor domains. SZ and ASD are well‐recognized as disorders with deficits in cognitive and sensory processing (Javitt & Freedman, 2015; Sinclair, Oranje, Razak, Siegel, & Schmid, 2017), representing by hyporesponsiveness or hyperresponsiveness to sensory stimuli (Acevedo, Aron, Pospos, & Jessen, 2018). Our results are compatible with the idea of relationships between cognitive/sensory processing and sensory/cerebellum abnormalities and provide further evidence supporting that the cerebellum and sensory systems are highly involved in cognition and behaviors. We speculated that the impaired cognitive and sensory processing in both diseases might be stem from deficits in sensory gating in patients. These deficits appear to be rooted in the similar unstable information flow between the cerebellum and sensorimotor cortex, as reflected by greater network reconfiguration between these functional domains.

On the other hand, sFNR at the ICN level shows divergence between SZ and ASD. Although the reconfiguration of thalamus‐connectivity increases in both SZ and ASD, it is only correlated with ADOS, not with the reasoning‐problem‐solving. Thalamus is a brain hub that receives and delivers information from‐to the whole‐brain. Atypical thalamocortical connectivity has been reported in both SZ and ASD, suggesting an overall impairment of thalamic pathways in two diseases (Andreasen, Paradiso, & O'Leary, 1998; Cerliani et al., 2015). Our findings are consistent with these observations but provide additional information that the thalamus alteration in SZ and ASD might underly different mechanisms, given that it is only associated with autistic traits in ASD, not with cognitive deficits in SZ.

The identified correlations between network reconfiguration and cognition/symptoms in this study are not voodoo due to the following reasons. First, we did not calculate the mean of those high correlations resulted from the nonindependent analysis, which is assumed as the major biased analysis that produces high correlations (Vul et al., 2009). Second, when performing the correlation analysis, we carefully controlled for age, gender, site, and diagnosis to make sure the results are not biased by these potential confounding effects. Our results were also replicated by using only the adult subjects from the ABIDE1 dataset. Third, we repeated our analysis using different window sizes (16–24 TRs) and the correlations were consistently observed within a wide range of window sizes.

### 4.4 Limitation and future direction

There are several limitations to this study. First, the drowsiness levels of participants might a potential confounding effect on the group differential functional network variability (Allen et al., 2018). Patients with SZ and ASD may have different levels of vigilance during the resting‐state scan, which might introduce unpredictable influence on our observations. Future studies can include cardiac, respiratory, and eye‐tracking techniques to monitor vigilance conditions during the scans. The medication history would be another confounding effect of the analysis. In our present study, we correlated the abnormal dynamic features in SZ and chlorpromazine, an antipsychotic medication used to treat psychotic disorders such as SZ and there is no significant correlation between them. More analysis on the influence of medication would be favored in our future studies.

Second, our study used two independent datasets that are collected from different databases. They have significantly different gender proportions and age ranges. Although the analysis was carefully performed on the patients and controls respectively, these confounding effects such as age, gender, and scanners might still potentially influence the results. In future work, our results should be validated by using the dataset with HCs, ASD patients, and SZ patients' data matched by age, gender, and scanners. We can also make direct comparisons between SZ and ASD, which will help to better elucidate the relationship between them.

Third, our study only focused on the behavioral and cognitive deficits in ASD and SZ. In addition to these deficits, impairments in motor function are also identified in ASD and SZ patients, which are typically interpreted as secondary phenomena for diagnosis (Huston, Shakow, & Riggs, 1937; Jansiewicz et al., 2006; Manschreck, 1981; Miller, Chukoskie, Zinni, Townsend, & Trauner, 2014; Shakow & Huston, 1936). Motor deficits and their relationship with abnormal dynamic functional connectivity as well as the treatment effects could be an interesting point and should be evaluated by future studies with the motor performance recorded.

It can be also an interesting point to investigate the transient heterogeneity/homogeneity of functional connectivity within a functional organization with atypical temporal patterns. In the future, by combining with the clustering‐based analysis (Allen et al., 2014; Allen et al., 2018; Fu et al., 2018; Fu et al., 2019), we can investigate whether a functional organization with atypical sFNR is associated with the transient increased or decreased functional connectivity within this functional organization in a specific brain state.

## CONFLICT OF INTEREST

The authors declare no conflict of interests.

## AUTHOR CONTRIBUTIONS

**Zening Fu**, **Yuhui Du**, and **Vince D. Calhoun** designed the study. **Zening Fu**, **Jing Sui**, and **Godfrey D. Pearlson** analyzed and interpreted the data. **Zening Fu**, **Jessica A. Turner**, **Michal Assaf**, **Godfrey D. Pearlson**, and **Vince D. Calhoun** wrote the paper. All authors revised the manuscript.

## Supporting information


**Appendix** **S1**: Supporting Information

## Data Availability

Data and MATLAB codes of this study can be obtained from the corresponding author with a reasonable request.
