# Supplementary material for: Dynamic functional network reconfiguration underlying the pathophysiology of schizophrenia and autism spectrum disorder
Source: Hum Brain Mapp. 2020 Sep 23;42(1):80–94. doi: 10.1002/hbm.25205 (PMC7721229; doi:10.1002/hbm.25205)
Supplement: Supplementary file 1 — Appendix S1: Supporting Information [file HBM-42-80-s001.docx]

Supplementary Materials for “Dynamic Functional Network Reconfiguration Underlying the Pathophysiology of Schizophrenia and Autism Spectrum Disorder”

# Comparing Individual Masks and Group Mask for fMRI Subject Selection

Whether subjects have good normalization to the template is important for the independent component analysis (ICA). A method based on the comparison of the individual mask and the group mask was introduced for subject selection. This method was applied to each dataset respectively. First, based on the first fMRI time volume, we calculated the individual mask for each subject by setting voxels which are greater than 90% of the whole brain mean to 1. Next, we computed a group mask by setting voxels which are included in more than 90% of the subjects to 1. For each subject, we then calculated the spatial correlations between the group mask and the individual mask. The spatial correlations were calculated using voxels within the top 10 slices of the mask, within the bottom 10 slices of the mask and within the whole mask, resulting in three correlation values for each subject. If a subject has correlations larger than 0.75 for the top 10 slices, larger than 0.55 for the bottom 10 slices, and larger than 0.8 for the whole mask, we include this subject for further ICA analysis. This ensures we have high-quality mask and fMRI data for all individuals and this approach has worked well for us in previous studies.

# Intrinsic Connectivity Networks (Peak Coordinates and Spatial Maps)

Peak Coordinates of Intrinsic Connectivity Networks (ICNs)

| **ICNs** | **X** | **Y** | **Z** |
| --- | --- | --- | --- |
| **Sub-cortical domain (SC)** | | | |
| Caudate (69) | 6.5 | 10.5 | 5.5 |
| Subthalamus/hypothalamus (53) | -2.5 | -13.5 | -1.5 |
| Putamen (98) | -26.5 | 1.5 | -0.5 |
| Caudate (99) | 21.5 | 10.5 | -3.5 |
| Thalamus (45) | -12.5 | -18.5 | 11.5 |
| **Auditory domain (AUD)** | | | |
| Superior temporal gyrus ([STG], 21) | 62.5 | -22.5 | 7.5 |
| Middle temporal gyrus ([MTG], 56) | -42.5 | -6.5 | 10.5 |
| **Sensorimotor domain (SM)** | | | |
| Postcentral gyrus ([PoCG], 3) | 56.5 | -4.5 | 28.5 |
| Left postcentral gyrus ([L PoCG], 9) | -38.5 | -22.5 | 56.5 |
| Paracentral lobule ([ParaCL], 2) | 0.5 | -22.5 | 65.5 |
| Right postcentral gyrus ([R PoCG], 11) | 38.5 | -19.5 | 55.5 |
| Superior parietal lobule ([SPL], 27) | -18.5 | -43.5 | 65.5 |
| Paracentral lobule ([ParaCL], 54) | -18.5 | -9.5 | 56.5 |
| Precentral gyrus ([PreCG], 66) | -42.5 | -7.5 | 46.5 |
| Superior parietal lobule ([SPL], 80) | 20.5 | -63.5 | 58.5 |
| Postcentral gyrus ([PoCG], 72) | -47.5 | -27.5 | 43.5 |
| **Visual domain (VS)** | | | |
| Calcarine gyrus ([CalcarineG], 16) | -12.5 | -66.5 | 8.5 |
| Middle occipital gyrus ([MOG], 5) | -23.5 | -93.5 | -0.5 |
| Middle temporal gyrus ([MTG], 62) | 48.5 | -60.5 | 10.5 |
| Cuneus (15) | 15.5 | -91.5 | 22.5 |
| Right middle occipital gyrus ([R MOG], 12) | 38.5 | -73.5 | 6.5 |
| Fusiform gyrus (93) | 29.5 | -42.5 | -12.5 |
| Inferior occipital gyrus ([IOG], 20) | -36.5 | -76.5 | -4.5 |
| Lingual gyrus ([LingualG], 8) | -8.5 | -81.5 | -4.5 |
| Middle temporal gyrus ([MTG], 77) | -44.5 | -57.5 | -7.5 |
| **Cognitive-control domain (CC)** | | | |
| Inferior parietal lobule ([IPL], 68) | 45.5 | -61.5 | 43.5 |
| Insula (33) | -30.5 | 22.5 | -3.5 |
| Superior medial frontal gyrus ([SMFG], 43) | -0.5 | 50.5 | 29.5 |
| Inferior frontal gyrus ([IFG], 70) | -48.5 | 34.5 | -0.5 |
| Right inferior frontal gyrus ([R IFG], 61) | 53.5 | 22.5 | 13.5 |
| Middle frontal gyrus ([MiFG], 55) | -41.5 | 19.5 | 26.5 |
| Inferior parietal lobule ([IPL], 63) | -53.5 | -49.5 | 43.5 |
| Left inferior parietal lobue ([R IPL], 79) | 44.5 | -34.5 | 46.5 |
| Supplementary motor area ([SMA], 84) | -6.5 | 13.5 | 64.5 |
| Superior frontal gyrus ([SFG], 96) | -24.5 | 26.5 | 49.5 |
| Middle frontal gyrus ([MiFG], 88) | 30.5 | 41.5 | 28.5 |
| Hippocampus ([HiPP], 48) | 23.5 | -9.5 | -16.5 |
| Left inferior parietal lobule ([L IPL], 81) | 45.5 | -61.5 | 43.5 |
| Middle cingulate cortex ([MCC], 37) | -15.5 | 20.5 | 37.5 |
| Inferior frontal gyrus ([IFG], 67) | 39.5 | 44.5 | -0.5 |
| Middle frontal gyrus ([MiFG], 38) | -26.5 | 47.5 | 5.5 |
| Hippocampus ([HiPP], 83) | -24.5 | -36.5 | 1.5 |
| **Default-mode domain (DM)** | | | |
| Precuneus (32) | -8.5 | -66.5 | 35.5 |
| Precuneus (40) | -12.5 | -54.5 | 14.5 |
| Anterior cingulate cortex ([ACC], 23) | -2.5 | 35.5 | 2.5 |
| Posterior cingulate cortex ([PCC], 71) | -5.5 | -28.5 | 26.5 |
| Anterior cingulate cortex ([ACC], 17) | -9.5 | 46.5 | -10.5 |
| Precuneus (51) | -0.5 | -48.5 | 49.5 |
| Posterior cingulate cortex ([PCC], 94) | -2.5 | 54.5 | 31.5 |
| **Cerebellar domain (CB)** | | | |
| Cerebellum ([CB], 13) | -30.5 | -54.5 | -42.5 |
| Cerebellum ([CB], 18) | -32.5 | -79.5 | -37.5 |
| Cerebellum ([CB], 4) | 20.5 | -48.5 | -40.5 |
| Cerebellum ([CB], 7) | 30.5 | -63.5 | -40.5 |


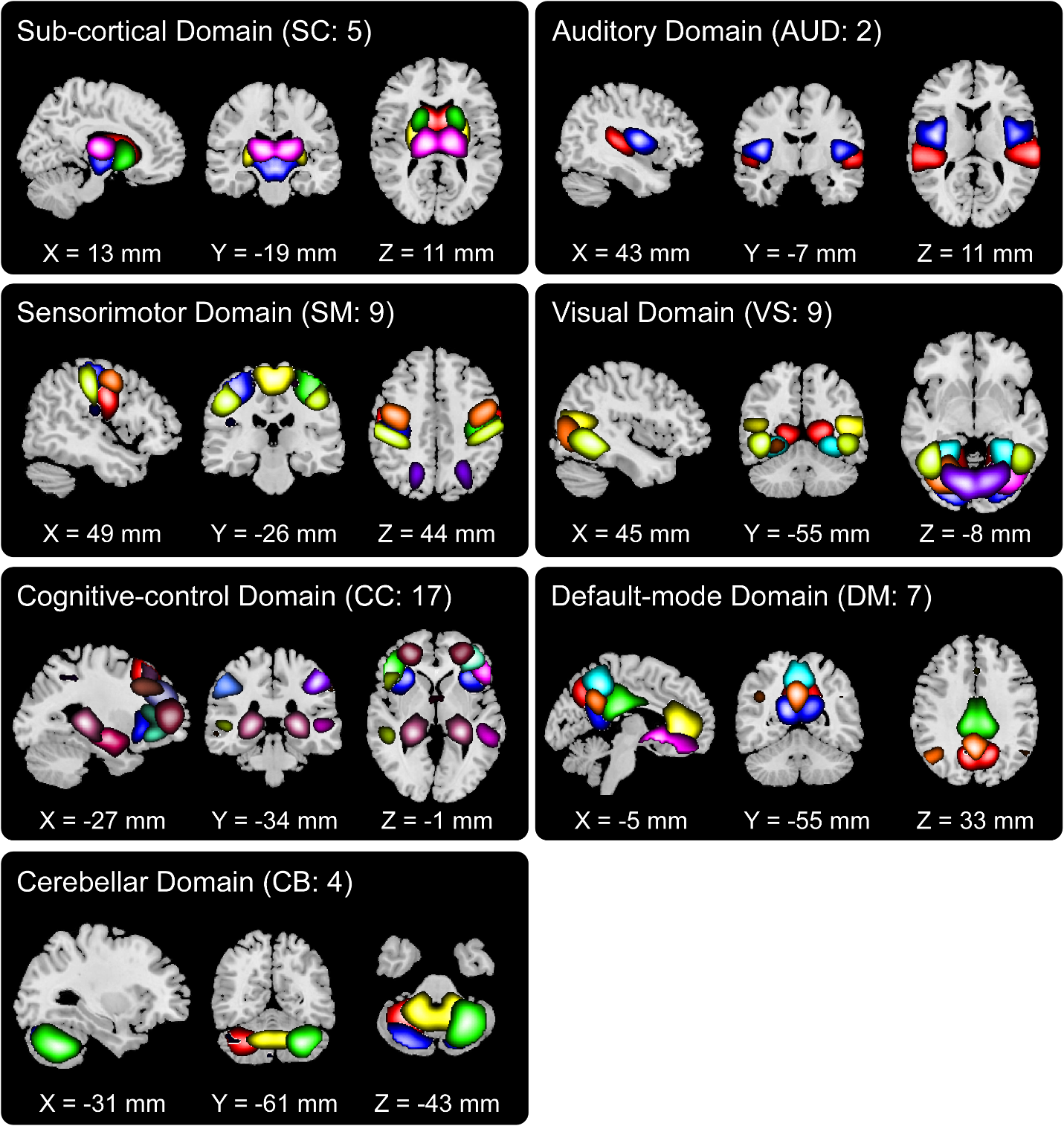


**Spatial maps of intrinsic connectivity networks (ICNs) extracted from the genomics superstruct project (GSP) dataset.** ICNs are arranged into 7 functional domains according to their functional and anatomical prior knowledge. There are 5 ICNs within subcortical domain (SC), 2 ICNs within auditory domain (AUD), 9 ICNs within sensorimotor domain (SM), 9 ICNs within visual domain (VS), 17 ICNs within cognitive-control domain (CC), 7 ICNs within default-mode domain (DM), and 4 ICNs within cerebellar domain (CB).


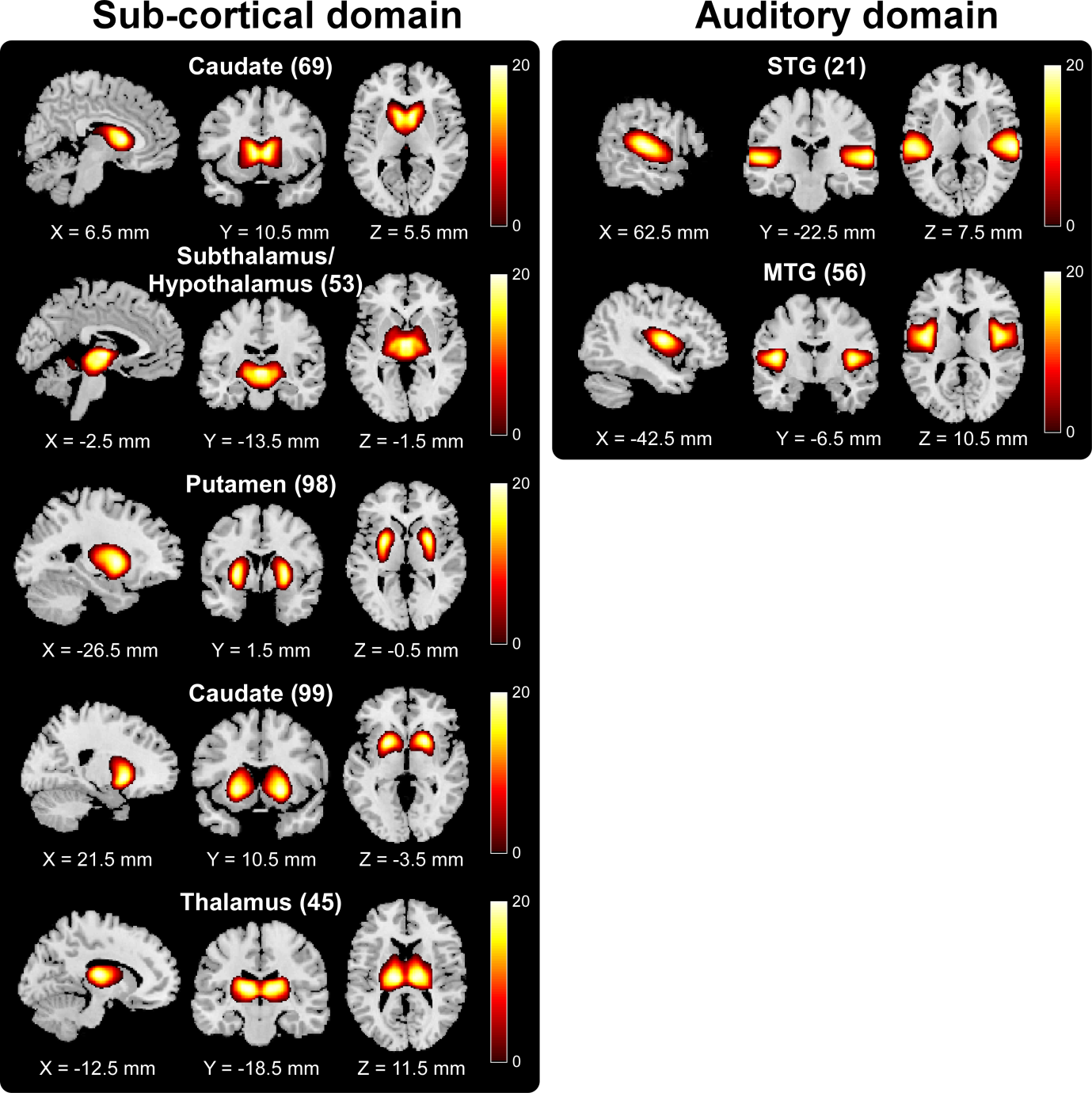


**Spatial map of each intrinsic connectivity networks (ICNs).** ICNs are divided into the 7 domains shown in Figure 1 and are thresholded at |*t*|>10, where one-sample t-statistics have been computed across the single-subject spatial maps. Sagittal, coronal, and axial slices are shown at the maximal t-statistic for clusters larger than 3 cm^3^.


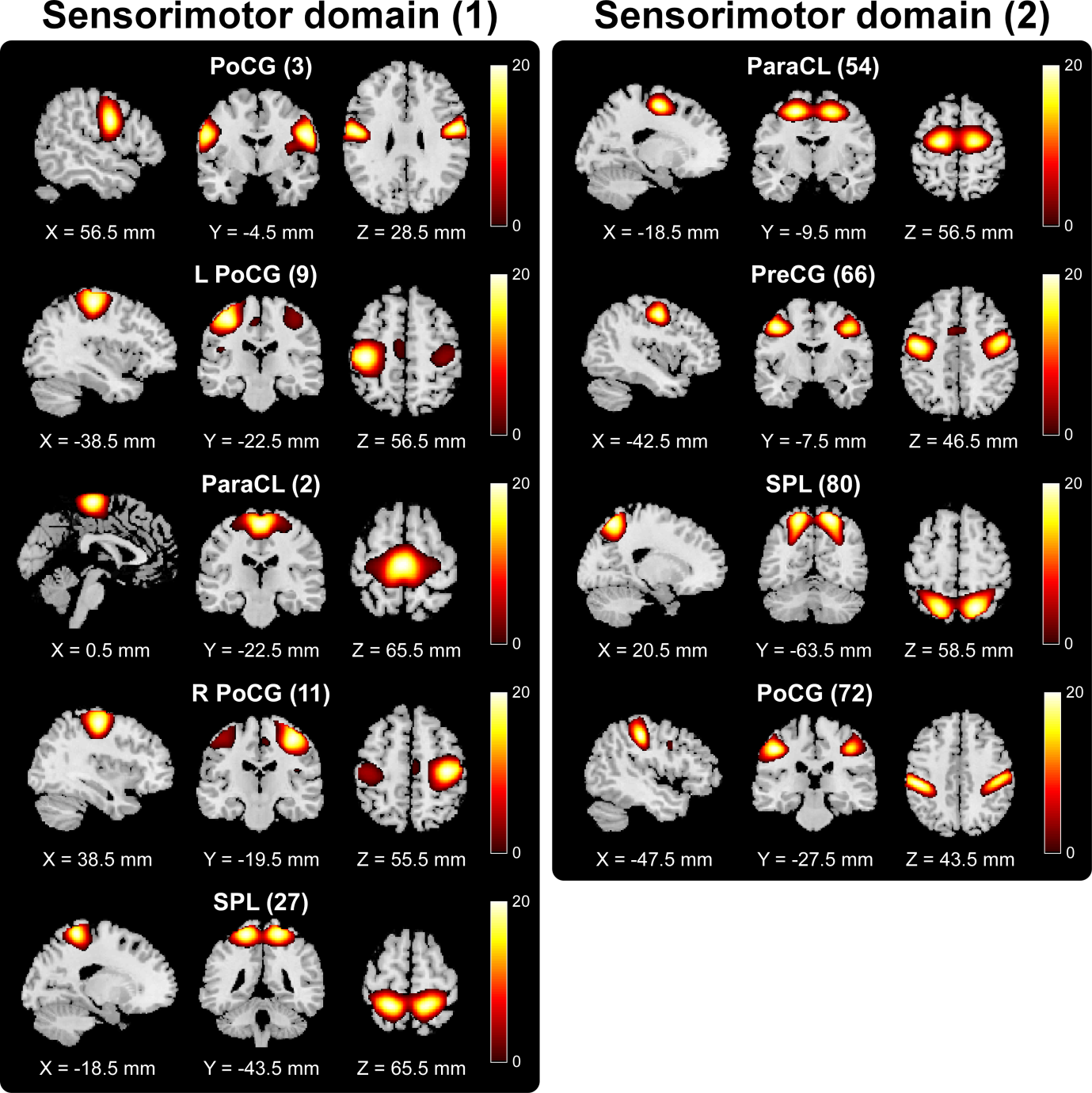


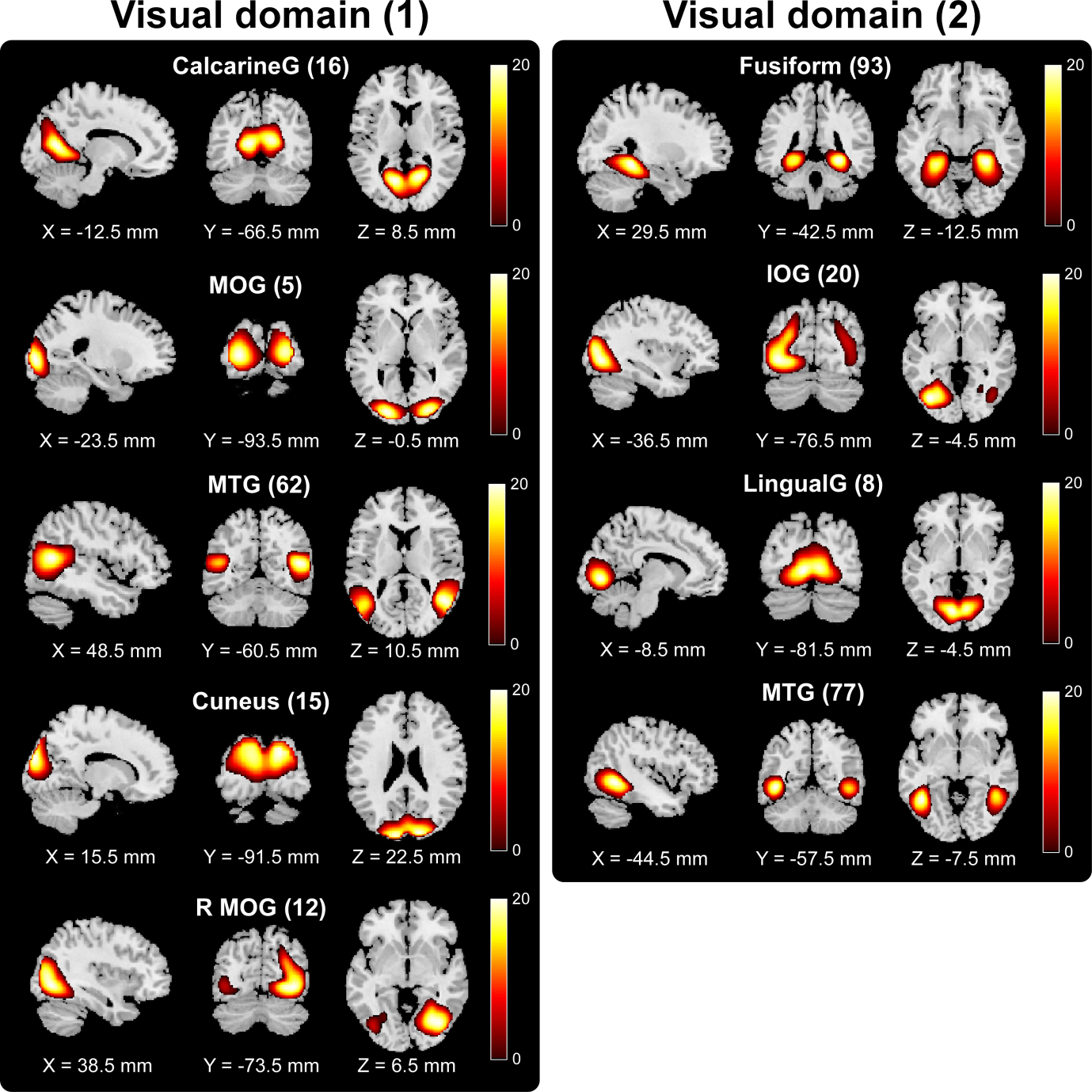


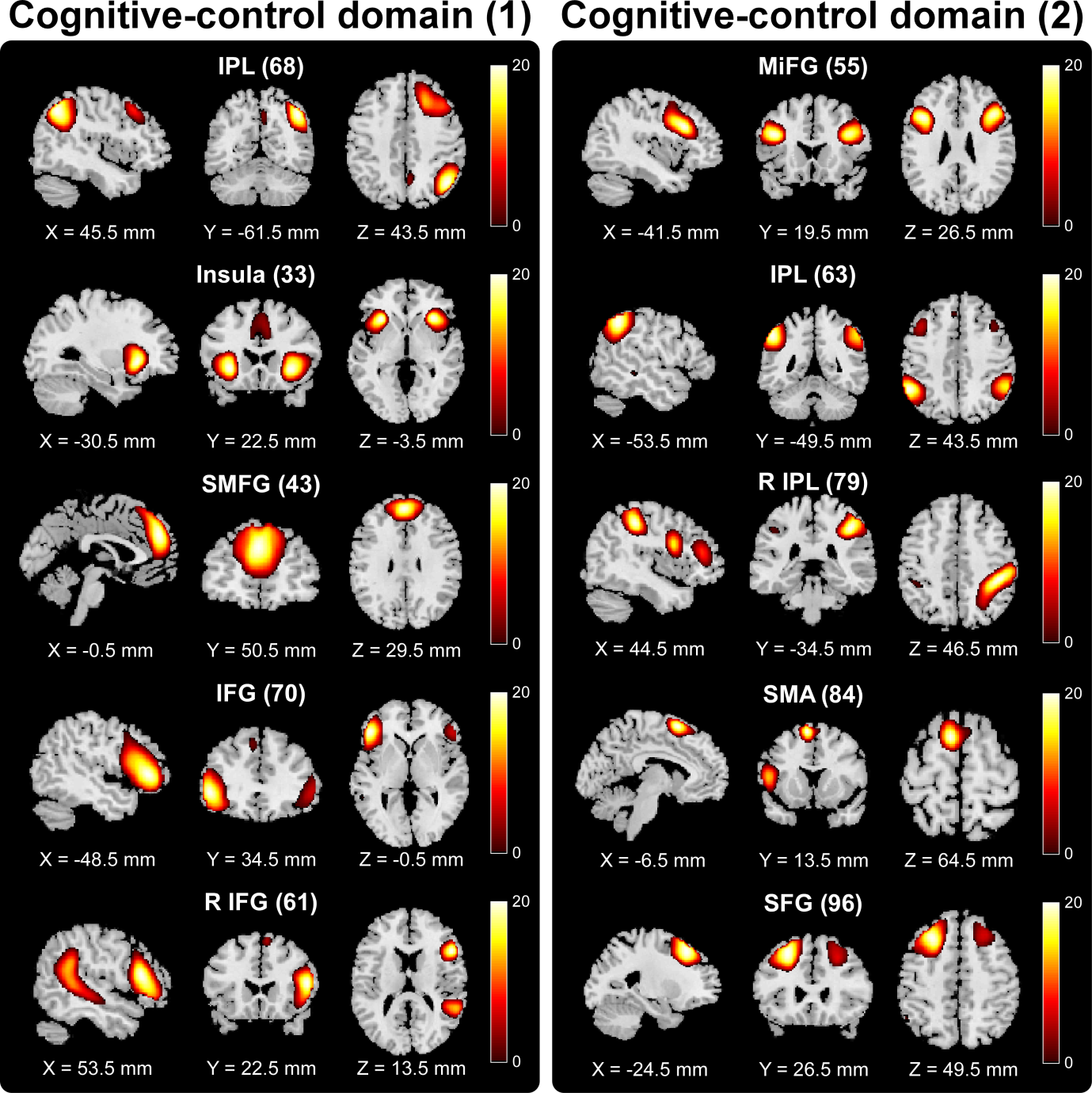


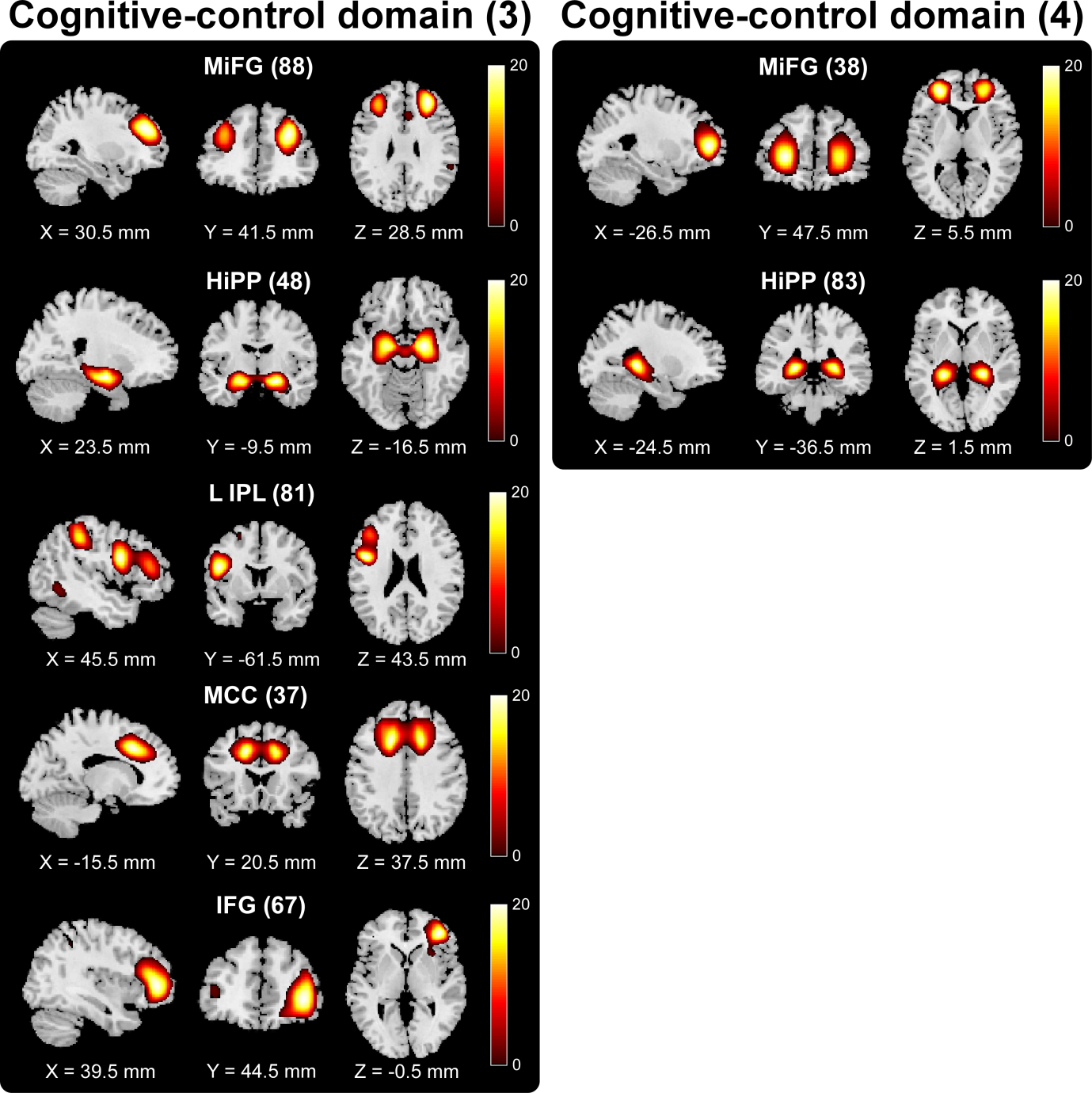


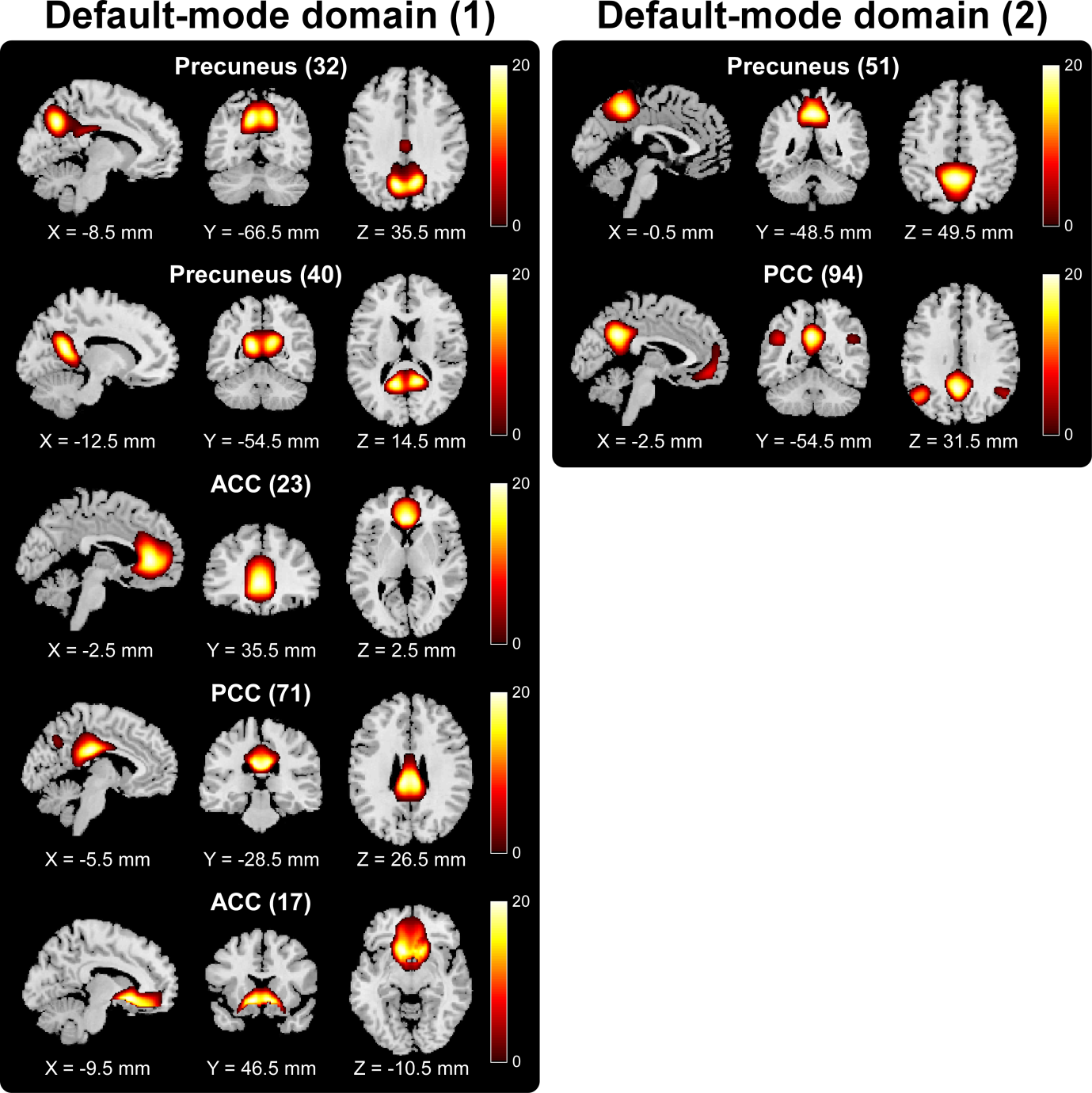


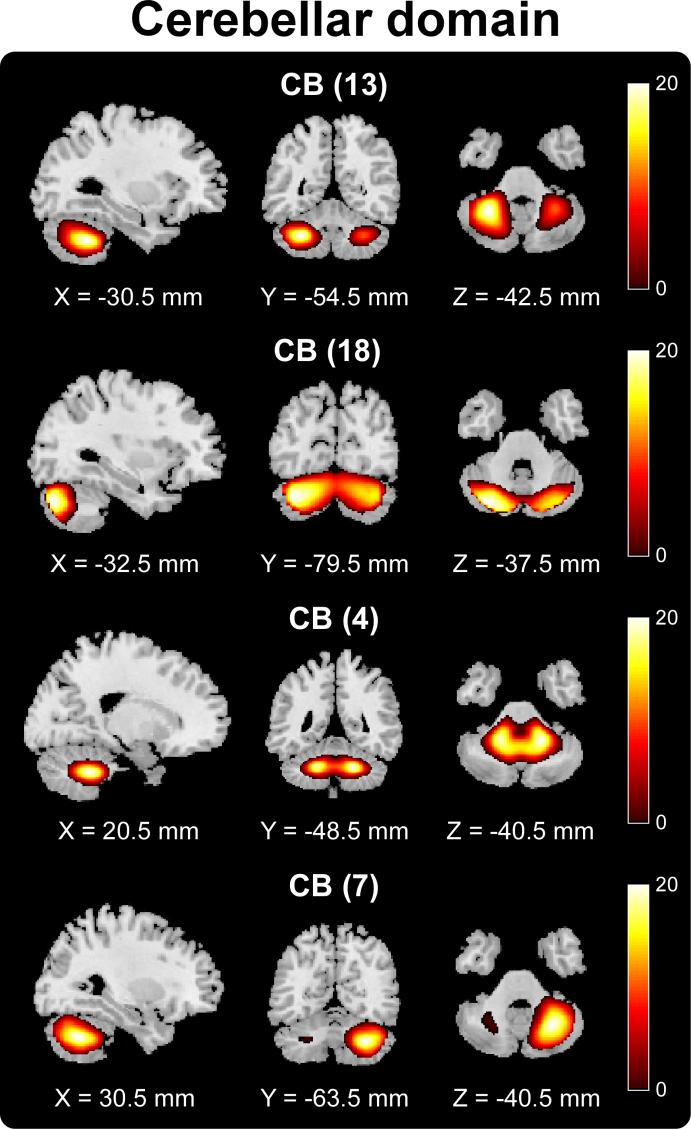


# Results of Abnormal sFNR in SZ and the Associations between sFNR and Cognitive Deficits Controlling for Site

The FBIRN dataset is a dataset consisting of subjects with [schizophrenia](https://www.sciencedirect.com/topics/medicine-and-dentistry/schizophrenia) or [schizoaffective disorder](https://www.sciencedirect.com/topics/medicine-and-dentistry/schizoaffective-disorder) along with healthy comparison subjects. Although the data were collected from multiple sites, they were scanned using the same structural and [functional scan parameters](http://fcon_1000.projects.nitrc.org/indi/abide/scan_params/KKI/rest.pdf) (e.g. data length, TR/TE, and slice order). Such a harmonized protocol will result in less heterogeneity across subjects.

To further show that the site information will not bias our findings, we repeated the statistical analysis between HCs and SZ using the GLM by controlling for the site effect in the FBIRN data. The SZ data used in this study were scanned from 7 different sites and therefore we created a 311 × 6 site array as the input covariate in the GLM analysis. The other potential confounding effects, including age, gender, and diagnosis (only for the association analysis) were also taken into account. Compared with HC, SZ has increased sFNR of the whole brain (p = 9.08e-5). Greater sFNR are identified between SC and SM/VS/CB domains, between CB and SM/CC/DM domains, and within CB domains. Thalamus, STG, SPL, calcarine, and cerebellum also show greater sFNR in SZ. The overall results (Table S2~S3, Figure S3) are consistent with those in the main manuscript.

Results of FBIRN controlling for site (Whole-brain/Between-domain/Within-domain)

| **Statistics** | Whole-brain | **Between-domain/Within-domain** | | | | | | |
| --- | --- | --- | --- | --- | --- | --- | --- | --- |
|  |  | SC vs. SM | SC vs. VS | SC vs. CB | CB vs. SM | CB vs. CC | CB vs. DM | CB vs. CB |
| Controlling for site | SZ > HC  (p = 9.08e-5) | SZ > HC  (p = 8.95e-6) | SZ > HC  (p = 1.06e-6) | SZ > HC  (p = 2.02e-7) | SZ > HC  (p = 2.66e-12) | SZ > HC  (p = 1.46e-5) | SZ > HC  (p = 1.05e-5) | SZ > HC  (p = 8.67e-5) |

Results of FBIRN controlling for site (ICNs)

| **Statistics** | **ICNs** | | | | |
| --- | --- | --- | --- | --- | --- |
|  | Thalamus | STG | SPL | Calcarine | Cerebellum |
| Controlling  for site | SZ > HC  (p = 7.34e-9) | SZ > HC  (p = 1.08e-4) | SZ > HC  (p = 2.75e-4) | SZ > HC  (p = 5.23e-5) | SZ > HC  (p = 5.10e-7) |


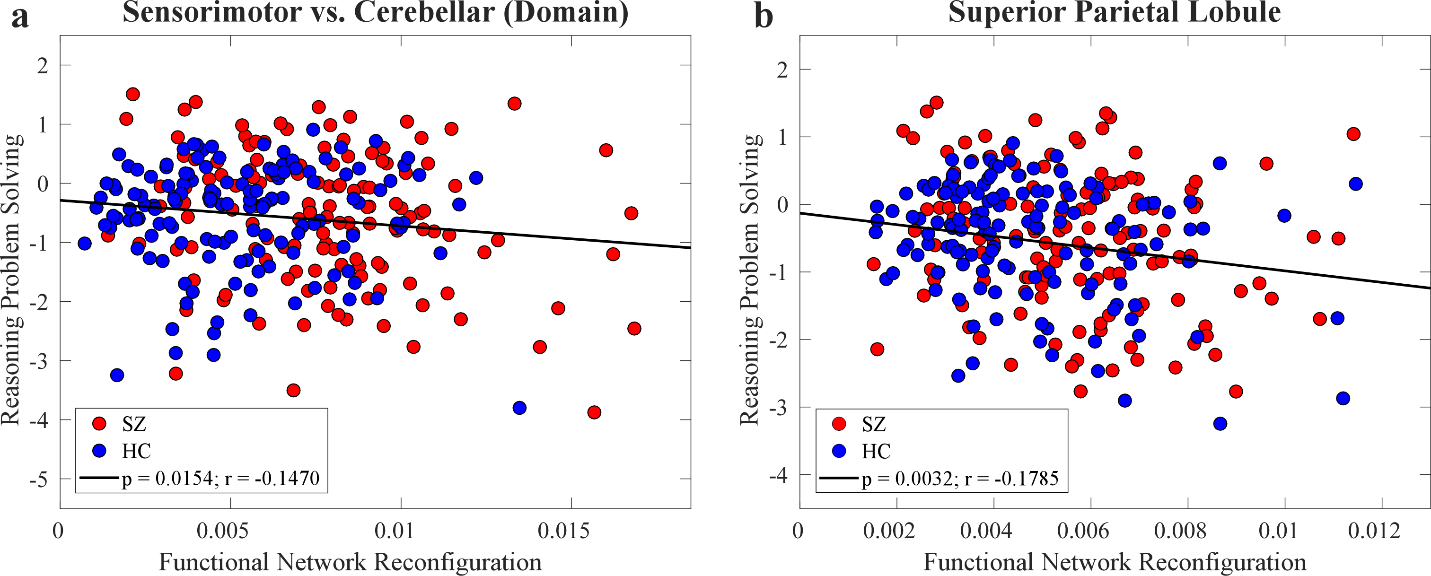


**Correlations between sFNR and reasoning-problem-solving score controlling for site.** Two sFNR calculations show negative correlations with reasoning-problem-solving: (A) sFNR between SM and CB domains in SZ; (B) sFNR of SPL in SZ. Each dot represents the value of each subject and the black line represents the relationship between sFNR and score.

# Results of Abnormal sFNR and the Associations between sFNR and Cognitive Deficits and Autistic Traits by Different Window Sizes

Compared with static metrics, dynamic functional connectivity analysis is relatively sensitive to the subject data quality and the parameter selection, such as the window size. Although studies have also provided strong evidence for the neuronal origin of temporal fluctuations of sliding window functional connectivity in hemodynamics, reliability and reproducibility of the sliding window approach is still a challenging issue in dynamic functional connectivity studies. To show the robustness and validity of our results, we used the sliding window approach with different window sizes (range from 16 TRs to 24 TRs: 32 s to 48 s) to estimate dFNC between ICNs. Then we calculated sFNR of different functional organizations and performed the GLM analysis to investigate the abnormal sFNR in SZ and ASD with different window sizes. The results are displayed in Table S4~S7, which are highly consistent with the results by 20 TRs in the main manuscript. We also investigated the associations between abnormal sFNR and cognitive deficits in SZ and autistic traits in ASD using GLM analysis. Similarly, the score of reasoning-problem-solving is negatively correlated with the sFNR between CB and DM domains and the sFNR of SPL in SZ data, while the ADOS is negatively correlated with the sFNR between CB and DM domains and the sFNR of thalamus in ASD data (Figure S4~S5).

Results of FBIRN by different window sizes (Whole-brain/Between-domain/Within-domain)

| **Statistics** | Whole-brain | **Between-domain/Within-domain** | | | | | | |
| --- | --- | --- | --- | --- | --- | --- | --- | --- |
|  |  | SC vs. SM | SC vs. VS | SC vs. CB | CB vs. SM | CB vs. CC | CB vs. DM | CB vs. CB |
| Window size  (16 TRs) | SZ > HC  (p = 2.76e-4) | SZ > HC  (p = 3.30e-5) | SZ > HC  (p = 3.61e-6) | SZ > HC  (p = 4.73e-6) | SZ > HC  (p = 1.66e-12) | SZ > HC  (p = 1.53e-4) | SZ > HC  (p = 1.75e-5) | SZ > HC  (p = 0.0026) |
| Window size  (18 TRs) | SZ > HC  (p = 1.26e-4) | SZ > HC  (p = 2.21e-5) | SZ > HC  (p = 2.61e-6) | SZ > HC  (p = 1.03e-6) | SZ > HC  (p = 2.40e-12) | SZ > HC  (p = 6.53e-5) | SZ > HC  (p = 1.66e-5) | SZ > HC  (p = 3.01e-4) |
| **Window size**  **(20 TRs)** | **SZ > HC**  **(p = 1.26e-4)** | **SZ > HC**  **(p = 1.19e-5)** | **SZ > HC**  **(p = 1.28e-6)** | **SZ > HC**  **(p = 2.67e-7)** | **SZ > HC**  **(p = 3.84e-12)** | **SZ > HC**  **(p = 2.05e-5)** | **SZ > HC**  **(p = 1.45e-5)** | **SZ > HC**  **(p = 1.31e-4)** |
| Window size  (22 TRs) | SZ > HC  (p = 8.29e-5) | SZ > HC  (p = 1.25e-5) | SZ > HC  (p = 1.39e-6) | SZ > HC  (p = 2.38e-7) | SZ > HC  (p = 1.60e-11) | SZ > HC  (p = 2.19e-5) | SZ > HC  (p = 1.32e-5) | SZ > HC  (p = 8.26e-5) |
| Window size  (24 TRs) | SZ > HC  (p = 1.32e-4) | SZ > HC  (p = 1.62e-5) | SZ > HC  (p = 1.79e-6) | SZ > HC  (p = 2.56e-7) | SZ > HC  (p = 6.37e-11) | SZ > HC  (p = 4.74e-5) | SZ > HC  (p = 1.13e-5) | SZ > HC  (p = 1.96e-4) |

Results of FBIRN by different window sizes (ICNs)

| **Statistics** | **ICNs** | | | | |
| --- | --- | --- | --- | --- | --- |
|  | Thalamus | STG | SPL | Calcarine | Cerebellum |
| Window size  (16 TRs) | SZ > HC  (p = 8.17e-9) | SZ > HC  (p = 5.29e-4) | SZ > HC  (p = 0.0013) | SZ > HC  (p = 2.53e-4) | SZ > HC  (p = 3.45e-6) |
| Window size  (18 TRs) | SZ > HC  (p = 5.98e-9) | SZ > HC  (p = 3.97e-4) | SZ > HC  (p = 8.89e-4) | SZ > HC  (p = 1.62e-4) | SZ > HC  (p = 1.72e-6) |
| **Window size**  **(20 TRs)** | **SZ > HC**  **(p = 1.09e-8)** | **SZ > HC**  **(p = 1.30e-4)** | **SZ > HC**  **(p = 3.73e-4)** | **SZ > HC**  **(p = 5.24e-5)** | **SZ > HC**  **(p = 1.01e-6)** |
| Window size  (22 TRs) | SZ > HC  (p = 8.68e-8) | SZ > HC  (p = 7.35e-5) | SZ > HC  (p = 1.73e-4) | SZ > HC  (p = 2.54e-5) | SZ > HC  (p = 2.45e-6) |
| Window size  (24 TRs) | SZ > HC  (p = 3.34e-7) | SZ > HC  (p = 5.40e-5) | SZ > HC  (p = 8.95e-5) | SZ > HC  (p = 1.52e-5) | SZ > HC  (p = 6.45e-6) |

Results of ABIDE by different window sizes (Whole-brain/Between-domain/Within-domain)

| **Statistics** | Whole-brain | **Between-domain/Within-domain** | | | | |
| --- | --- | --- | --- | --- | --- | --- |
|  |  | SC vs. CB | SM vs. AUD | SM vs. VS | SM vs. CB | CC vs. DM |
| Window size  (16 TRs) | ASD > HC  (p = 4.64e-4) | ASD > HC  (p = 0.0032) | ASD > HC  (p = 0.0115) | ASD > HC  (p = 0.0024) | ASD > HC  (p = 1.14e-7) | ASD > HC  (p = 0.0194) |
| Window size  (18 TRs) | ASD > HC  (p = 3.70e-4) | ASD > HC  (p = 0.0021) | ASD > HC  (p = 0.0058) | ASD > HC  (p = 0.0018) | ASD > HC  (p = 1.56e-7) | ASD > HC  (p = 0.0136) |
| **Window size**  **(20 TRs)** | **ASD > HC**  **(p = 4.04e-4)** | **ASD > HC**  **(p = 0.0020)** | **ASD > HC**  **(p = 0.0046)** | **ASD > HC**  **(p = 0.0015)** | **ASD > HC**  **(p = 3.49e-7)** | **ASD > HC**  **(p = 0.0085)** |
| Window size  (22 TRs) | ASD > HC  (p = 5.59e-4) | ASD > HC  (p = 0.0029) | ASD > HC  (p = 0.0091) | ASD > HC  (p = 0.0034) | ASD > HC  (p = 1.34e-6) | ASD > HC  (p = 0.0086) |
| Window size  (24 TRs) | ASD > HC  (p = 5.74e-4) | ASD > HC  (p = 0.0019) | ASD > HC  (p = 0.0240) | ASD > HC  (p =0.0070) | ASD > HC  (p = 1.75e-6) | ASD > HC  (p = 0.0100) |

Results of ABIDE by different window sizes (ICNs)

| **Statistics** |  | **ICNs** | | | | | |
| --- | --- | --- | --- | --- | --- | --- | --- |
|  | Thalamus | | PreCG | IOG | IPL | PCC | Cerebellum |
| Window size  (16 TRs) | ASD > HC  (p = 0.0013) | | ASD > HC  (p = 0.0135) | ASD > HC  (p = 0.0168) | ASD > HC  (p = 0.0039) | ASD > HC  (p = 0.0024) | ASD > HC  (p = 0.0014) |
| Window size  (18 TRs) | ASD > HC  (p = 0.0022) | | ASD > HC  (p = 0.0076) | ASD > HC  (p = 0.0119) | ASD > HC  (p = 0.0021) | ASD > HC  (p = 0.0022) | ASD > HC  (p = 0.0021) |
| **Window size**  **(20 TRs)** | **ASD > HC**  **(p = 0.0063)** | | **ASD > HC**  **(p = 0.0019)** | **ASD > HC**  **(p = 0.0098)** | **ASD > HC**  **(p = 7.32e-4)** | **ASD > HC**  **(p = 0.0030)** | **ASD > HC**  **(p = 0.0025)** |
| Window size  (22 TRs) | ASD > HC  (p = 0.0114) | | ASD > HC  (p = 6.34e-4) | ASD > HC  (p = 0.0122) | ASD > HC  (p = 6.24e-4) | ASD > HC  (p = 0.0063) | ASD > HC  (p = 0.0043) |
| Window size  (24 TRs) | ASD > HC  (p = 0.0046) | | ASD > HC  (p = 4.66e-4) | ASD > HC  (p = 0.0131) | ASD > HC  (p = 5.56e-4) | ASD > HC  (p = 0.0117) | ASD > HC  (p = 0.0040) |


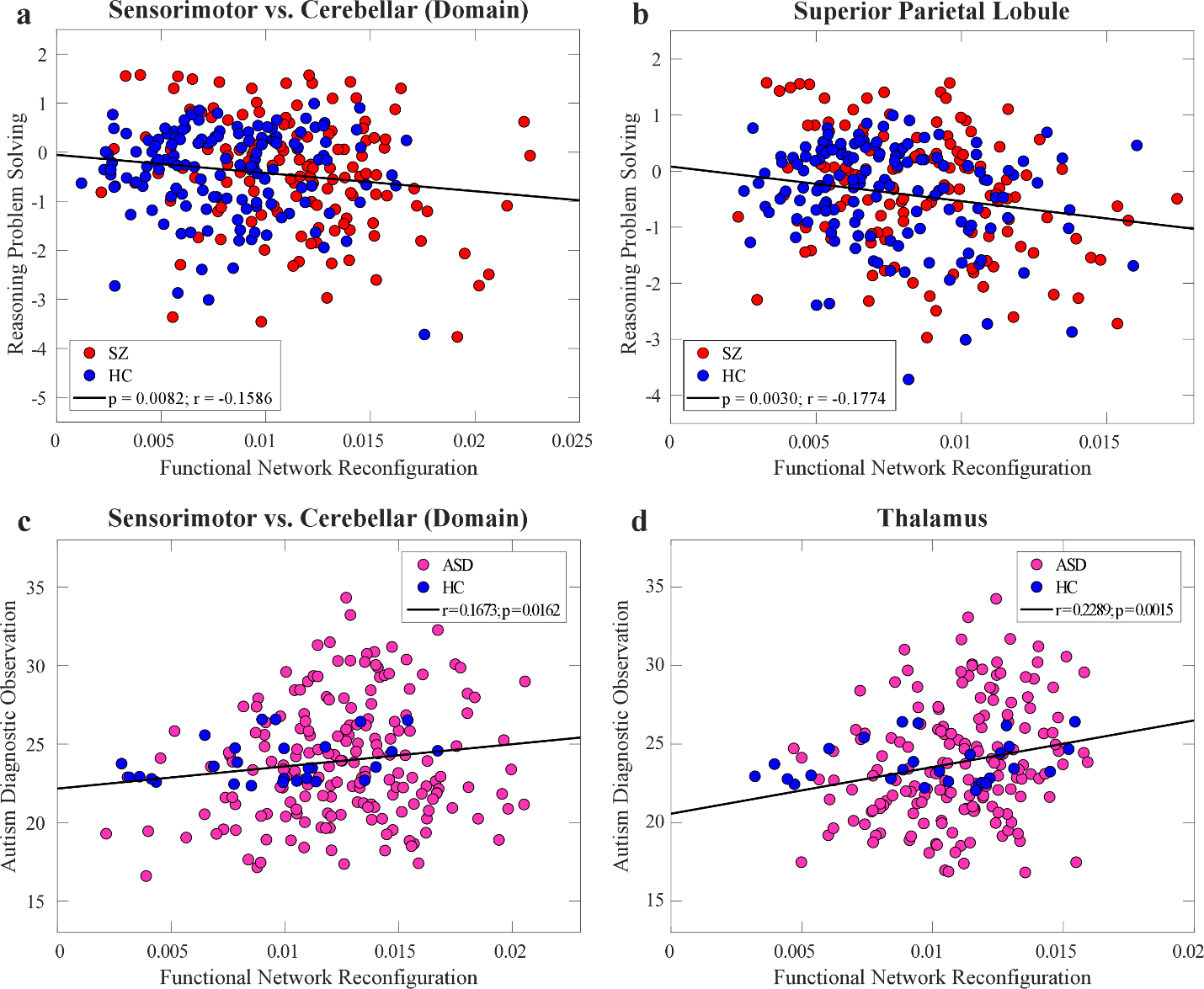


**Correlations between sFNR and reasoning-problem-solving score/ADOS by window size of 16 TRs.** Two sFNR calculations show negative correlations with reasoning-problem-solving and two sFNR calculations show positive correlations with ADOS: (A) sFNR between SM and CB domains in SZ; (B) sFNR of SPL in SZ; (C) sFNR between SM and CB domains in ASD; (D) sFNR of thalamus in ASD. Each dot represents the value of each subject and the black line represents the relationship between sFNR and score.


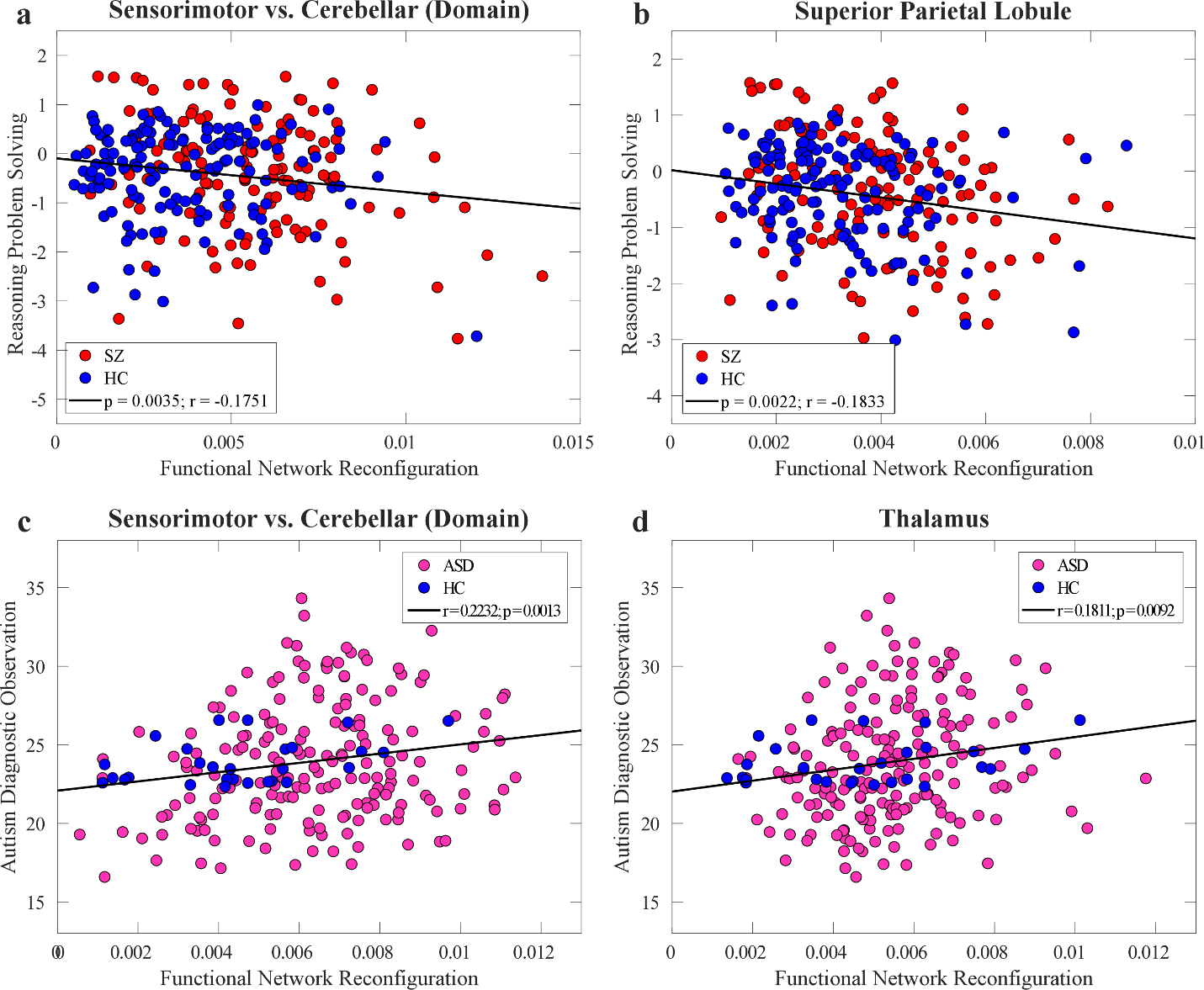


**Correlations between sFNR and reasoning-problem-solving score/ADOS by window size of 24 TRs.** Two sFNR calculations show negative correlations with reasoning-problem-solving and two sFNR calculations show positive correlations with ADOS: (A) sFNR between SM and CB domains in SZ; (B) sFNR of SPL in SZ; (C) sFNR between SM and CB domains in ASD; (D) sFNR of thalamus in ASD. Each dot represents the value of each subject and the black line represents the relationship between sFNR and score.

# Results of Abnormal sFNR in ASD and the Associations between sFNR and ADOS using Adult Subjects

The SZ data and ASD data used in this study were collected from two different databases, resulting in significantly different age ranges. Considering that age could be a potential confounding effect, we carefully performed the statistical analysis by controlling for age in the examination of group differences and probing the associations between sFNR and cognitive deficits and symptoms. To further prove that the observed results are not biased by the brain maturation, we included only the adult subjects in the ABIDE data (age >= 20 years) and repeated the analysis between ASD and HCs. 142 adult subjects were included in the analysis and the GLM was performed by controlling for age, gender, site, and diagnosis (for association analysis only). The results are provided in Table S8~S9 and Figure S6. The majority of our findings in the main manuscript can still hold, including the increased sFNR of the whole-brain, increased sFNR between SM and CB domains, and the positive associations between abnormal sFNR and ADOS.

Results of ABIDE using only adult subjects (Whole-brain/Between-domain/Within-domain)

| **Statistics** | Whole-brain | **Between-domain/Within-domain** | | | | |
| --- | --- | --- | --- | --- | --- | --- |
|  |  | SC vs. CB | SM vs. AUD | SM vs. VS | SM vs. CB | CC vs. DM |
| Age >= 20 | ASD > HC  (p = 0.0188) | ASD > HC  (p = 9.05e-4) | ASD > HC  (p = 0.0456) | ASD > HC  (p =0.0789) | ASD > HC  (p = 0.0118) | ASD > HC  (p = 0.0420) |

Results of ABIDE using only adult subjects (ICNs)

| **Statistics** |  | **ICNs** | | | | | |
| --- | --- | --- | --- | --- | --- | --- | --- |
|  | Thalamus | | PreCG | IOG | IPL | PCC | Cerebellum |
| Age >= 20 | ASD > HC  (p = 0.0362) | | ASD > HC  (p = 0.2313) | ASD > HC  (p = 0.0395) | ASD > HC  (p = 0.0117) | ASD > HC  (p = 0.3539) | ASD > HC  (p = 0.0101) |


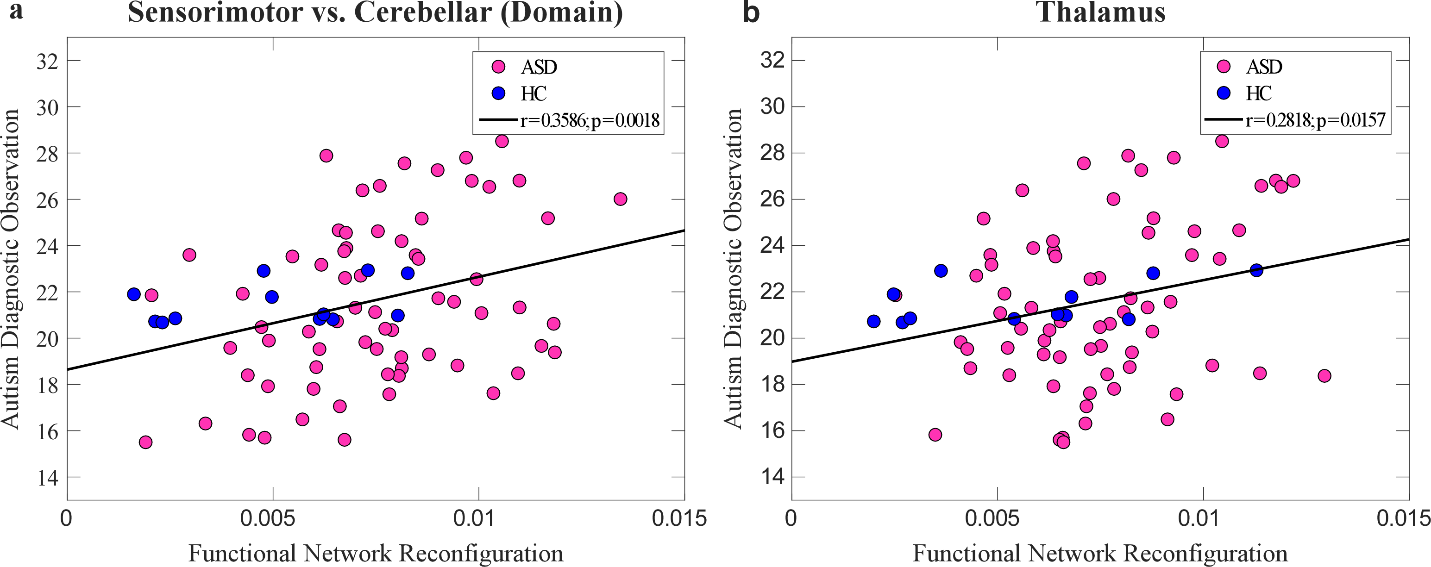


**Correlations between sFNR and ADOS in adult subjects from ABIDE.** Two sFNR calculations show negative correlations with ADOS: (A) sFNR between SM and CB domains in ASD; (B) sFNR of thalamus in ASD. Each dot represents the value of each subject and the black line represents the relationship between sFNR and ADOS.
